# Conserved pathogenesis of ancestral and contemporary Oropouche virus strains in a murine pregnancy model

**DOI:** 10.1038/s41467-026-72711-2

**Published:** 2026-05-12

**Authors:** Krista B. Gunter, James M. Bowen, Andrew T. Clarke, Melanie McFarlane, Dorcus C. A. Omoga, Stephanie Pozuelos, Henry Giesel, Curtis Witt, Lisa M. Rogers, David M. Aronoff, Andrew M. Lunel, Jay Vornhagen, Benjamin Brennan, Natasha L. Tilston

**Affiliations:** 1 Department of Microbiology and Immunology, Indiana University School of Medicine, Indianapolis, IN USA; 2 MRC-University of Glasgow Centre for Virus Research, Glasgow, UK; 3 Department of Medicine, Indiana University School of Medicine, Indianapolis, IN USA; 4 Department of Pediatrics, Herman B Wells Center for Pediatric Research, Indiana University School of Medicine, Indianapolis, IN USA

**Keywords:** Viral pathogenesis, Viral evolution

## Abstract

Oropouche virus (OROV) is an emerging orthobunyavirus responsible for widespread outbreaks across South and Central America. Recent reports of congenital disease have raised urgent concerns regarding the potential risk of OROV infection during pregnancy. Here, we establish an in vivo murine model of OROV vertical transmission using the ancestral (prototype) strain BeAn19991 in immunocompetent C57BL/6 J mice. We demonstrate that OROV robustly replicates in maternal tissues and efficiently infects the placenta. Complementary studies in human trophoblast-derived cell lines demonstrate conserved placental tropism across both the ancestral strain and a contemporary (outbreak) isolate, supporting the translational relevance of our findings. Notably, comparison of ancestral and contemporary viruses indicates that placental infection is not a recently acquired property of OROV. Further, offspring born to infected dams exhibit maternally derived neutralizing antibodies and transient protection upon postnatal challenge. Together, these findings, considered alongside emerging epidemiological evidence, identify pregnancy as a critical context for OROV infection and underscore the need to evaluate risks to pregnant individuals in endemic regions.

## Abstract (web summary)

Oropouche virus (OROV) is an emerging virus in Latin America. Here the authors show in a mouse model that OROV infects the placenta and can reach the fetus during pregnancy, a conserved feature across ancestral and recent strains that highlights emerging risks to maternal and fetal health.

## Introduction

Oropouche virus (OROV) has emerged as a defining example of how ecological change, vector expansion, and viral evolution can rapidly reshape the epidemiology of neglected arboviruses. Since late 2022, OROV transmission has intensified across the Americas, with sustained outbreaks persisting through 2025 in countries including Brazil, Cuba, and Panama^1–3^. These trends prompted epidemiological alerts from regional and international public health agencies, reflecting growing recognition that OROV is no longer a sporadic or geographically constrained pathogen^4–10^.

Oropouche virus (OROV), the etiologic agent of Oropouche fever, is an orthobunyavirus primarily transmitted by *Culicoides paraensis* midges and has circulated endemically in parts of South America since the 1960s^11^. However, the recent outbreak is unprecedented in both scale and geographic distribution. For the first time, OROV has spread beyond its traditional range into new countries such as Cuba, which reported 626 confirmed and over 24,000 suspected cases in 2024 alone^12,13^. This expansion resulted in imported cases in non-endemic regions, including the United States, Canada, the United Kingdom, and continental Europe^1^. Notably, we identified *Culicoides sonorensis*, a widespread North American midge species, as a competent vector for OROV^14^, raising the possibility of local transmission if the virus is introduced. Together with broader environmental and climate-related factors influencing vector ecology^15,16^, these observations heighten concerns about the potential for future OROV establishment in North America^17–19^.

In parallel with its geographic spread, the clinical profile associated with OROV infection has expanded during recent outbreaks to include pregnancy-associated infections^20–22^, neurological complications^1^, and fatalities^1,23^. These observations may reflect a combination of increased case detection, changes in population exposure and potential viral genetic variation. Once considered a self-limiting febrile illness, OROV is now associated with a broader spectrum of clinical outcomes, highlighting the growing public health threat posed by historically overlooked arboviruses and underscoring the urgent need to reassess OROV’s pathogenic potential.

OROV is a tri-segmented, single-stranded, negative-sense RNA virus (*Orthobunyavirus oropoucheense*, *Peribunyaviridae*, *Bunyavirales*)^24^. Its genome comprises three segments: the S segment encodes the nucleoprotein (N) and the non-structural protein NSs in overlapping open reading frames; the M segment encodes a glycoprotein precursor that is co-translationally cleaved into glycoproteins Gn and Gc, as well as the NSm protein; and the L segment encodes the RNA-dependent RNA polymerase (RdRp). Each segment is flanked by untranslated regions (UTRs) that harbor signals essential for replication, transcription, and packaging^25^. OROV’s segmented genome facilitates reassortment, a process in which genome segments are exchanged during co-infection with other orthobunyaviruses, thereby accelerating evolutionary change^11,26^. Multiple naturally occurring reassortants, such as Iquitos virus, Madre de Dios virus, and Perdões virus, have emerged through inter-species reassortment events, often involving an unidentified donor of the M segment^27^. Although rare, such events can result in substantial shifts in virulence or host range^28,29^. By contrast, intra-species reassortment among circulating OROV strains appears to occur more frequently and has been implicated in the genetic diversification of strains associated with the recent outbreak^3^. These strains/isolates likely represent complex reassortment events among divergent S, M, and L segment lineages^3,30^, and while such intra-species reassortment events are often phenotypically neutral, the number of congenital infections associated with OROV cases during this outbreak has raised concerns that these genetic exchanges may contribute to altered pathogenesis^1^. The extent to which genome segment exchange influences OROV virulence, particularly in the context of maternal-fetal transmission, remains poorly understood and represents a critical gap in our knowledge.

Here, we developed a murine model of OROV infection during pregnancy using the ancestral strain BeAn19991 to investigate the virus’s capacity for vertical transmission. Through a combination of timed gestational infections in immunocompetent mice and parallel studies in human placental cell lines, we demonstrate that OROV can cross the maternal-fetal interface and infect the placental tissue. Importantly, our findings indicate that vertical transmission is not a feature unique to contemporary isolates but rather a conserved property of OROV biology that may have been underappreciated. Together, our results establish an immunocompetent model of OROV infection during pregnancy and provide evidence that supports the relevance of OROV infection to maternal-fetal health.

## Results

### OROV replicates efficiently in the liver and spleen of immunocompetent C57BL/6J mice

To establish a physiologically relevant model of OROV infection during pregnancy, we first assessed whether immunocompetent wild-type (WT) C57BL/6J mice support viral replication. Using our previously validated recombinant (*r*) reporter OROV (rOROVMZsG)^31^, which we demonstrated was lethal in type I interferon (IFN) receptor knock-out (IFNAR^−/−^) mice, we conducted a 14-day infection study (Supplemental Fig. 1a). Infected mice exhibited no overt clinical signs and gained weight comparably to animals inoculated with UV-inactivated virus, indicating minimal disease burden (Supplemental Fig. 1b). Quantification of viral RNA (vRNA) across tissues revealed OROV replication in the liver and spleen at early time points. At 5 days post-infection (dpi), liver vRNA levels ranged from below the limit of detection (LOD; ND) to 9.9 log_10_ RNA copies/g, while splenic vRNA ranged from ND to 9.1 log_10_ RNA copies/g (Supplemental Fig. 1c). By 7 dpi, vRNA levels had declined but remained detectable in a subset of animals, with liver and spleen vRNA from ND to 8.2 log_10_ RNA copies/g and ND to 7 log_10_ RNA copies/g, respectively. By 14 dpi, vRNA levels in both tissues were uniformly low, ranging from 5.2 to 6 log_10_ RNA copies/g in the liver and 5.2 to 6.6 log_10_ RNA copies/g in the spleen. In contrast, vRNA detection in heart, lung, and brain was sporadic and generally low across all time points, with most samples falling below LOD and occasional detection not exceeding ~5 – 6 log_10_ RNA copies/g.

Consistent with active viral replication, infectious virus was recovered from liver and spleen homogenates at 5 dpi, with productive infection confirmed by reporter virus detection in Vero E6 cells (Supplemental Fig. 1d). Collectively, these results demonstrate that rOROV establishes a productive, but self-limiting infection in immunocompetent WT C57BL/6J mice, with primary replication localized to the liver and spleen.

### OROV infects the placenta and crosses the maternal-fetal barrier in C57BL/6J mice

**Fig. 1:**
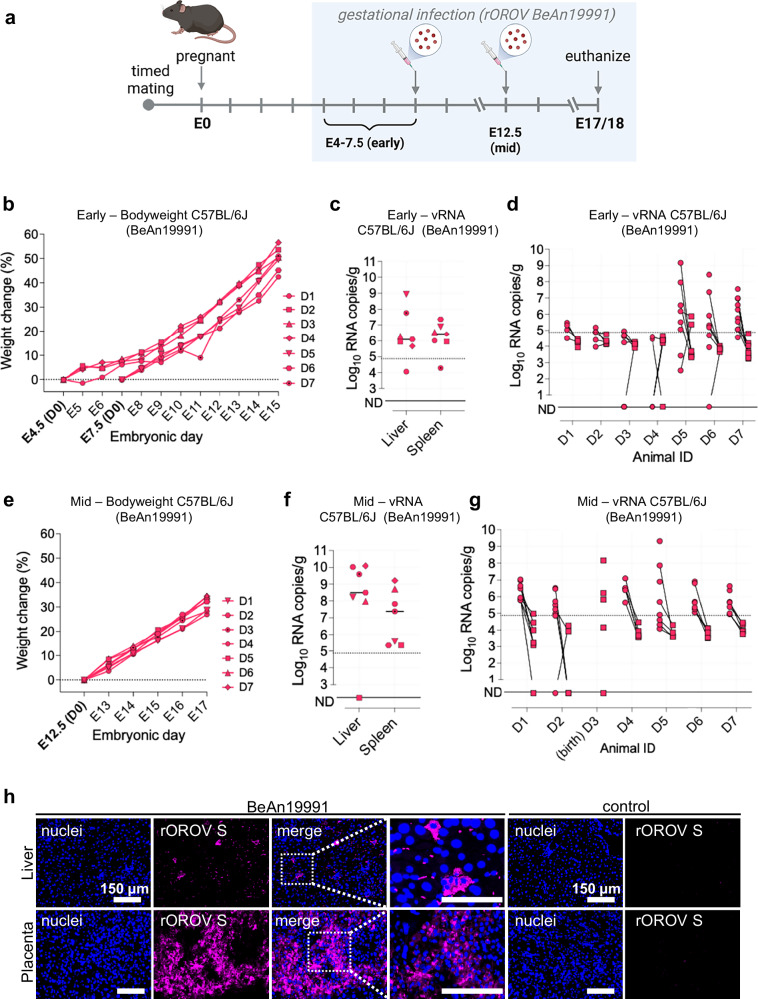
Infection with rOROV BeAn19991 leads to robust maternal infection and placental dissemination. **a** Schematic of experimental design (Created in BioRender. Tilston-lunel, N. (2026) https://BioRender.com/ ldb9voq). Six-week-old C57BL/6J female mice were housed with C57BL/6J male mice for 48 h to permit mating. Pregnant dams were infected SC with BeAn19991 at E4-7.5 (early gestation) or E12.5 (mid-gestation) and euthanized between E17–18. Percent weight gain of pregnant BeAn19991-infected dams following early gestation (**b**) or mid (**e**) gestation (*n* = 7 dams per group). vRNA levels in maternal liver and spleen at harvest, measured by RT-qPCR at early (**c**) or mid (**f**) gestation (*n* = 7 dams per group, each point represents an individual dam). vRNA levels in matched placentas (circles) and fetuses (squares) from each dam, quantified by RT-qPCR at early (**d**) or mid (**g**) gestation. Each point represents an individual placenta or fetus derived from independent pregnancies (*n* = 7 dams per group; placenta and fetus numbers vary). The dashed lines represent the limit of detection, ND = not detected. **h** HCR RNA-FISH detection of rOROV S segment vRNA (magenta) in maternal liver and placental sections from BeAn19991-infected dams. Infected hepatocytes (liver; top) exhibit cytoplasmic or perinuclear vRNA accumulation. Placental sections (bottom) reveal widespread infection in the labyrinth zone. Nuclei stained with Hoechst (blue). Scale bars as indicated. (EVOS M5000 imaging system, ThermoFisher). Source data are provided as Source Data file.

To investigate the potential for vertical transmission, timed-pregnant C57BL/6J dams were subcutaneously inoculated with the ancestral strain rOROV BeAn19991^32^ at two gestational time points, E7.5 and E12.5. Dams infected at E7.5 (Early gestation) or E12.5 (Mid gestation) were necropsied at E17.5, corresponding to 10 or 5 dpi, respectively (Fig. 1a). In both groups, dams gained weight steadily throughout pregnancy, with no evidence of fetal reabsorption or pregnancy loss (Fig. 1b, e and Supplemental Fig. 3a). Analysis of maternal tissues revealed high vRNA burdens in liver and spleen in both groups (Fig. 1c, f). Following early gestation infection, maternal liver and spleen vRNA levels ranged from 4.5 to 9 log_10_ RNA copies/g, whereas mid-gestation infection resulted in higher levels, ranging from ND to 10 log_10_ RNA copies/g, consistent with sampling near peak systemic replication as observed in non-pregnant mice (Supplementary Fig. 1c). In contrast, vRNA in control dams remained at or below LOD (Supplementary Fig. 3b).

To evaluate vertical transmission, we quantified vRNA in matched placentas (circles) and fetuses (squares) collected at necropsy. OROV vRNA in placental tissue was consistently detected in both early and mid-gestation groups with placental vRNA levels ranging from ND to 9.2 log_10_ RNA copies/g and ND to 7.9 log_10_ RNA copies/g, respectively (circles; Fig. 1d, g). In contrast, fetal tissues harbored detectable vRNA only in a subset of samples (squares; Fig. 1d, g). Gross fetal morphology appeared normal across all groups, with no overt developmental abnormalities observed at either gestational stage (Supplemental Fig. 2a). In mock-infected controls, vRNA levels in placental and fetal tissues were largely at or near the LOD, with no evidence of productive infection (Supplemental Fig. 3c).

To visualize vRNA, we applied a custom hybridization chain reaction (HCR) RNA-FISH assay targeting OROV S vRNA, as described in our recent study^33^. In infected maternal liver sections, we detected discrete foci of vRNA, with signal localized to the cytoplasm or perinuclear regions of hepatocytes, consistent with active viral replication (Fig. 1h). No vRNA signal was detected in liver sections from control animals. In placental sections from infected dams, widespread vRNA accumulation was observed throughout the labyrinth zone, a major site of maternal-fetal exchange, indicating robust replication at the maternal-fetal interface (Fig. 1h). In contrast, no signal was detected in placentas from mock-infected controls. The distribution of vRNA signal in maternal liver and the placental tissues indicates localized clusters of infection, consistent with productive viral replication. To determine whether placental vRNA reflected replication-competent virus, placental homogenates were used to inoculate Vero E6 cells. Immunofluorescence imaging at 24 h post-infection (hpi) revealed rOROV-positive foci in a subset of samples (Supplemental Fig. 2b), confirming the presence of infectious virus in some, but not all, placentas. Consistent with a recent study demonstrating that successful OROV isolation requires high viral burden^34^, recovery of infectious virus was inefficient when placental vRNA levels were low. Similarly, isolation of infectious virus from whole fetal homogenates was largely unsuccessful, likely due to low viral loads and reduced cell viability following tissue processing; however, replication-competent virus was recovered from one representative fetal sample, and this result was independently reproduced (Supplementary Fig. 2c).

Histopathological analysis of maternal liver revealed mild to moderate extramedullary hematopoiesis in rOROV-infected dams compared to the mock-infected controls, consistent with localized inflammatory responses to infection (Supplementary Fig. 2d). Collectively, these data demonstrate that the ancestral OROV strain BeAn19991 productively replicates in the placenta following both early- and mid-gestational infection. Although fetal infection was less frequent, the detection of vRNA in select fetal tissues and replication-competent virus in placentas confirms that BeAn19991 can breach the maternal-fetal barrier and establish infection at the maternal-fetal interface.

**Fig. 2:**
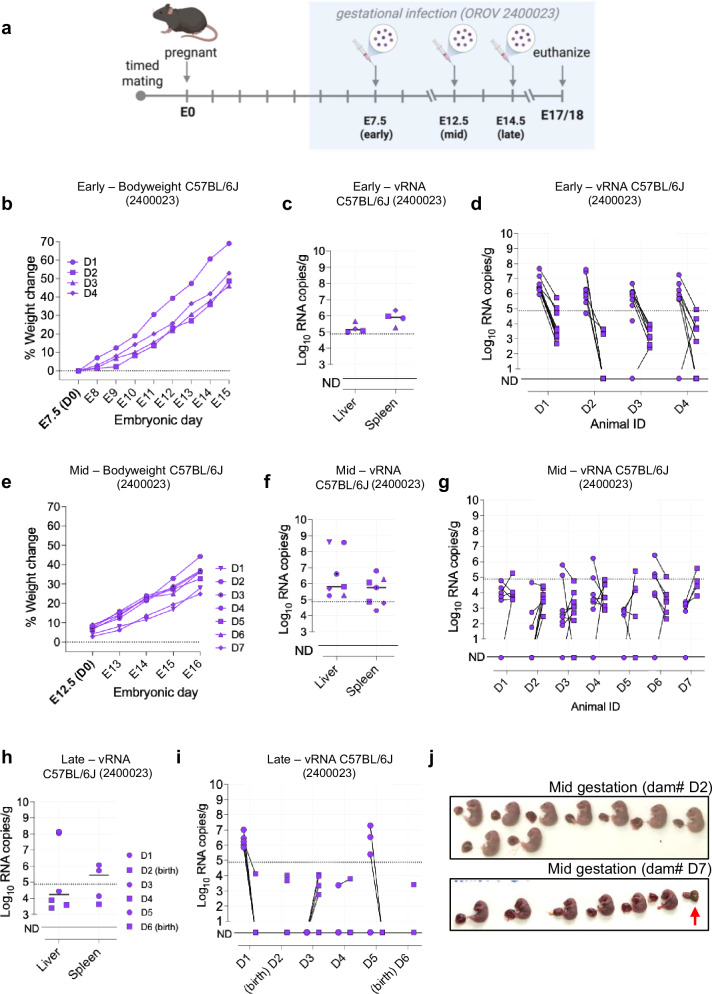
Infection with OROV 2400023 leads to robust placental infection at early gestational timepoint. **a** Schematic of experimental design. (Created in BioRender. Tilston-lunel, N. (2026) https://BioRender.com/ ldb9voq). Six-week-old C57BL/6J female mice were housed with C57BL/6J male mice for 48 h to permit mating. Pregnant dams were infected SC with OROV 2400023 at E7.5 (early gestation), E12.5 (mid-gestation), or E14.5 (late gestation), and euthanized between E17-18. Percent weight gain of pregnant 2400023-infected dams following early gestation (**b**) or mid (**e**) gestation. vRNA levels in maternal liver and spleen at harvest, measured by RT-qPCR at early (*n* = 4 dams) (**c**), mid (*n* = 7 dams) (**f**), or late (*n* = 6 dams) (**h**) gestation. Each point represents an individual dam. vRNA levels in matched placentas (circles) and fetuses (squares) from each dam, quantified by RT-qPCR at early (*n* = 4 dams) (**d**), mid (*n* = 7 dams) (**g**), or late (*n* = 6 dams) (**i**) gestation. Each point represents an individual placenta or fetus derived from independent pregnancies. The dashed lines represent the limit of detection, ND = not detected. **j** Representative gross fetal morphology at harvest (E17–18) from dams infected at mid-gestation (D2 and D7). A single fetus from dam D7 exhibited a gross morphological abnormality (red arrow).

Next, we evaluated whether a contemporary OROV isolate from the current outbreak crosses the maternal-fetal barrier more effectively than the ancestral strain. OROV strain 2400023 represents a contemporary outbreak-associated virus circulating in Cuba and is closely related to the Brazilian outbreak isolates, while harboring a limited number of coding differences across the glycoprotein and NSs genes, enabling comparison between ancestral and contemporary OROV strains in the context of this study. OROV strain 2400023 was used to infect timed-pregnant C57BL/6J dams at early (E7.5) and mid (E12.5) gestation similar to BeAn19991 with an additional late gestation (E14.5) time point (Fig. 2a). Across all infection time points, dams gained weight steadily throughout pregnancy, with no evidence of fetal resorption or pregnancy loss (Fig. 2b, e).

Following early gestation infection, vRNA was readily detected in maternal liver (5–5.7 log_10_ RNA copies/g) and spleen (5.3–6.3 log_10_ RNA copies/g) at necropsy (Fig. 2c), and placental vRNA levels (Fig. 2d, circles) consistently exceeded those in matched fetal tissues (Fig. 2d, squares). Placental vRNA levels at early gestation typically ranged from ND to 7.7 log_10_ RNA copies/g, whereas fetal vRNA levels were lower, frequently near or below the LOD. When directly compared with the ancestral strain BeAn19991, the frequency of placental infection at early gestation was significantly higher in the dams infected with OROV 2400023 (88.57% vs. 57.45%, χ²(1) = 9.387, *p* = 0.0022, odds ratio = 0.174, 95% CI [0.06, 0.50]). Furthermore, overall placental vRNA levels tended to be higher in the 2400023-group than the BeAn19991-group (Mann–Whitney U = 300, *n*_1_ = 27, *p* = 0.0650, two-tailed; Supplemental Fig. 4). In contrast, mid-gestation infection with OROV 2400023 resulted in robust maternal infection (liver = 5.3 to 8.6 and spleen = 4.3 to 6.8 log_10_ RNA copies/g), but comparatively lower placental vRNA levels (ND to 6.4 log_10_ RNA copies/g; Fig. 2g; circles). Most fetal values clustered near the LOD (Fig. 2g; squares). Following late gestation infection, maternal liver and spleen vRNA levels were reduced relative to the earlier time points (liver = 3.2 to 8.1 and spleen = 3.6 to 6.1 log_10_ RNA copies/g), and several animals progressed to delivery prior to scheduled necropsy, limiting paired fetal sampling. Despite this, placental vRNA levels at this late gestation were higher than those observed at mid gestation, ranging from ND to 7.3 log_10_ RNA copies/g (Fig. 2h, i). Gross fetal morphology was largely normal across all groups, with no overt developmental abnormalities. However, one fetus at mid-gestation showed an abnormality (Fig. 2j; red arrow), and it is unclear whether this was directly due to OROV infection, as we were unable to isolate the virus from this fetus.

To further determine whether OROV can infect the fetus, we investigated whether impairing maternal type I IFN signaling would enhance susceptibility to vertical OROV transmission. Here, we performed timed matings between IFNAR^-/-^ females and WT C57BL/6J males to generate heterozygous pregnancies, as previously described^35,36^. Pregnant dams were then inoculated with rOROV BeAn19991 at E 7.5 (Supplemental Fig. 5a). Because IFNAR^-/-^ mice succumb to OROV infection by ∼4 - 7 dpi (occurring earlier at higher doses), dams were euthanized at E14.5 for tissue collection. All infected IFNAR^-/-^ dams developed overt clinical disease, including ruffled fur, hunched posture, and weight loss, and one dam succumbed to infection at E13.5. High levels of vRNA were detected in maternal liver and spleen, confirming robust systemic infection in the absence of type I IFN receptor signaling (Supplemental Fig. 5b). Analysis of placental and fetal tissues revealed widespread viral dissemination; all placentas from infected dams harbored high vRNA loads (4 to 9.1 log_10_ RNA copies/g), with consistently lower but detectable levels in matched fetal tissues (4 to 8.5 log_10_ RNA copies/g), indicating efficient maternal-fetal transfer of virus (Supplemental Fig. 5c). To confirm vRNA detection, infectious virus in the placentas was recovered at >5 log_10_ TCID_50_/ml in multiple samples (Supplemental Fig. 5d). Infectious virus was also recovered from fetal tissues across several litters, although at lower titers than those observed in placentas. Immunofluorescence staining of placental and fetal sections from dam D2 confirmed the presence of OROV antigen in both compartments (Supplemental Fig. 5e; representative placenta and fetus #5). Despite extensive placental infection and detectable fetal viral burden, gross fetal morphology appeared largely intact, with no overt resorption or malformations observed. Fetal weights were not significantly different between mock-infected and infected litters (Welch’s t-test, *p* = 0.0653; Supplemental Fig. 5f).

Together, these findings demonstrate that loss of maternal type I IFN signaling markedly enhances placental infection and fetal dissemination of OROV, establishing type I IFN as a key barrier limiting vertical transmission. Notably, this provides a mechanistic framework for our observations in immunocompetent C57BL/6J dams, where placental and fetal vRNA levels were detectable but variable and frequently low. In contrast, disruption of this pathway resulted in uniformly higher viral burdens and more consistent recovery of replication-competent virus. Collectively, these data indicate that maternal innate immune control and viral burden, rather than intrinsic viral incapacity, likely constrain the efficiency of OROV vertical transmission.

### OROV productively infects human placental trophoblast cell lines independent of strain

**Fig. 3:**
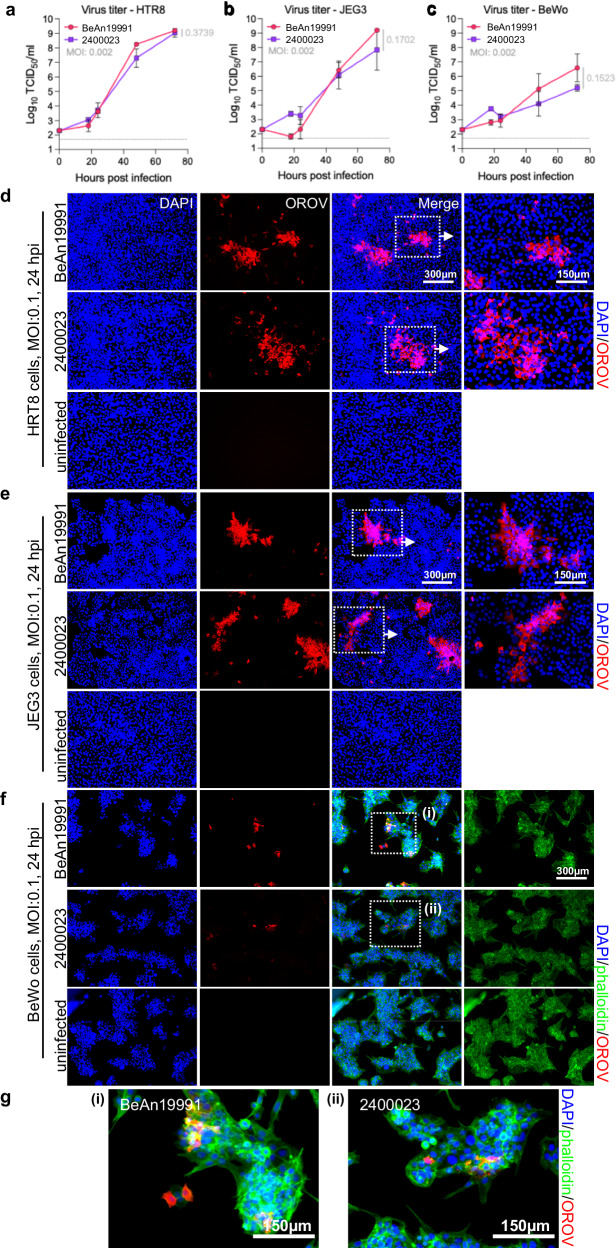
Ancestral and contemporary OROV replicate efficiently in human placental-derived cell lines. Growth kinetics of rOROV BeAn19991 and OROV 2400023 in human placental cell lines: HTR8 (**a**), JEG3 (**b**) and BeWo (**c**). Cells were infected at MOI 0.002, and supernatants were collected at 18, 24, 48, and 72 hpi. Data represent independent biological replicates (*n* = 3 independent infections, error bars ± SD). Statistical significance at the 72-h time point was determined using an unpaired two-tailed t test with false discovery rate (FDR) correction for multiple comparisons (two-stage step-up method of Benjamini, Krieger, and Yekutieli; *Q* = 1%). **d**–**g** Immunofluorescence imaging of infections in cells at MOI 0.1, stained with anti-OROV antibody (red) and DAPI (blue). Images are representative of *n* = 3 independent experiments with similar results. **g** High magnification fields of **f** Scale bars as indicated. BeWo cells were also stained with phalloidin (green). (EVOS M5000 imaging system, ThermoFisher).

To assess whether OROV placental tropism observed in vivo is conserved in human cells, we evaluated replication of the ancestral strain rOROV BeAn19991 and the contemporary outbreak isolate OROV 2400023 in three trophoblast-derived cell lines: HTR8 (first-trimester extravillous trophoblasts), BeWo, and JEG3 (choriocarcinoma-derived trophoblasts). Cells were infected at a low multiplicity of infection (MOI = 0.002), and viral titers in supernatants were quantified over time. Both strains productively infected all three cell lines, with robust replication kinetics observed across models (Fig. 3a–c). In HTR8 cells, viral titers increased rapidly, reaching peak levels of ~ 9–9.5 log_10_ TCID_50_/ml by 72 hpi for both BeAn19991 and 2400023 (Fig. 3a). In JEG3 and BeWo cells, replication proceeded more gradually but still achieved high titers, reaching approximately ~ 9 and 6.5–7.5 log_10_ TCID_50_/ml by 72 hpi, respectively (Fig. 3b, c). No statistically significant differences in peak viral titers were observed between strains in any cell line. Immunofluorescence analysis at 24 hpi confirmed widespread intracellular viral antigen accumulation in all three trophoblast models (Fig. 3d-g). In HTR8 and JEG3 cells, OROV antigen localized predominantly to cytoplasmic foci, forming discrete clusters consistent with active replication and cell-to-cell spread (Fig. 3d, e). In BeWo cells, Phalloidin staining was included to visualize actin architecture and cell boundaries, enabling assessment of cellular morphology during infection. This enabled us to accurately determine OROV antigen (Fig. 3f, g). No viral signal was detected in uninfected control cells. Together, these findings demonstrate that OROV efficiently infects and replicates in diverse human placental trophoblast cell types, and that this tropism is conserved between the ancestral BeAn19991 strain and a contemporary outbreak isolate.

### Contemporary OROV isolates exhibit increased replicative fitness

**Fig. 4:**
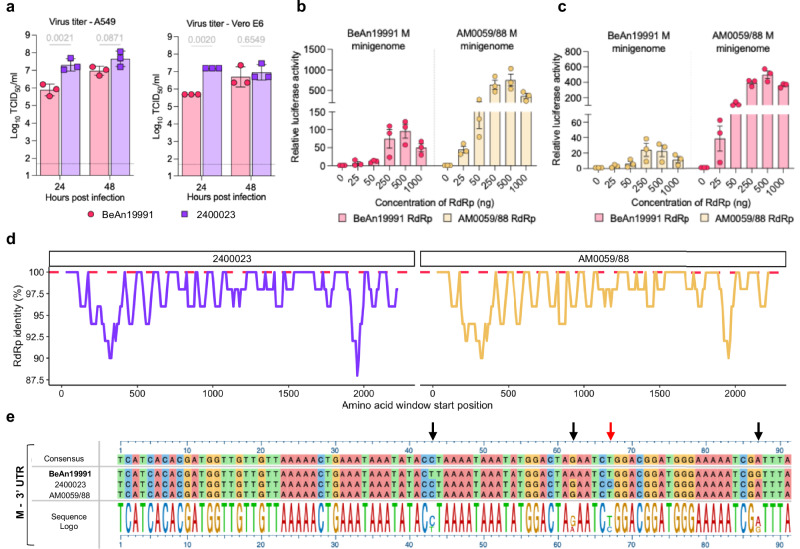
Contemporary OROV strains exhibit enhanced growth and altered polymerase activity. **a** Growth kinetics of rOROV BeAn19991 and OROV 2400023 in A549 and Vero E6 cells. Supernatants were collected at 24 and 48 hpi (MOI 0.1) and titrated by TCID_50_ assay. Data represent independent biological replicates (*n* = 3 independent infections) and are presented as mean ± SD. Statistical significance was determined using a two-way ANOVA followed by Šídák’s multiple comparisons test (two-sided, *α* = 0.05). **b**, **c** Cross-comparison of polymerase activity using BeAn19991 or AM0059/88 RdRp in M segment minigenome systems from BeAn19991 (red) and AM0059/88 (yellow). BSRT7/5 cells were transfected with an optimized ratio of N, and M-segment minigenome along with varying concentrations of RdRp expressing plasmids (Supplemental Fig. 6, and ref. ^42^). Luciferase activity was measured 24 hpt. Each point represents an independent transfection (*n* = 3 independent biological replicates per condition), and data are presented as mean ± SE. **d** Sliding window analysis of amino acid identity across the RdRp of OROV 2400023 (purple) and AM0059/88 (yellow) relative to BeAn19991 (dashed red). Identity values were plotted along the RdRp using a 50-amino-acid sliding window. **e** Alignment of the M segment 3′ UTR from BeAn19991, 2400023, and AM0059/88. Black arrows indicate substitutions between 2400023, AM0059/88 vs BeAn19991, and the red arrow denotes a unique insertion in 2400023. The sequence logo below shows nucleotide conservation.

To compare fitness differences between the ancestral and contemporary OROV, we first assessed viral replication in type I IFN-competent (A549) and IFN-deficient (Vero E6) cells. In both cell types, OROV BeAn19991 and the contemporary isolate OROV 2400023 reached comparable peak titers by 48 hpi, achieving approximately 7 log_10_ TCID_50_/ml (Fig. 4a). However, at 24 hpi, OROV 2400023 consistently replicated to a log higher than BeAn19991 in both A549 (mean 7.3 vs. 5.9 log_10_TCID_50_/ml; *p* = 0.0021) and Vero E6 (mean 7.2 vs. 5.7 log_10_ TCID_50_/m; *p* = 0.0020) cells, indicating a replicative advantage during early infection.

To extend this analysis to additional contemporary strains, we incorporated sequence data from the 2024 Brazilian isolates OROV AM0059 and AM0088, which possess identical M segments and are therefore referred to collectively as AM0059/88 (Drs. William M. de Souza, José Luiz Proenca-Modena, and Pritesh Lalwani; GenBank accession numbers PP992525-PP992530). As these virus isolates were unavailable from public repositories, we employed a minigenome system to assess transcriptional and replication efficiency under controlled conditions. Among the three genome segments, only the M segment contained complete 3′ and 5′ UTRs; therefore, an M-segment-based minigenome encoding a humanized *Renilla* luciferase reporter was generated. Luciferase activity from the AM0059/88 M minigenome was robust and increased in a dose-dependent manner with both N and RdRp expression, peaking at a 1:1 ratio (Supplemental Fig. 6). In contrast, the BeAn19991 M minigenome consistently exhibited lower activity across all conditions, with peak values ~10-fold lower (Fig. 4b, c). Fig. 4b compares each M-segment minigenome paired with its cognate polymerase, whereas Fig. 4c directly tests polymerase dependence by pairing both M-segment minigenomes with the heterologous RdRp. The persistence of enhanced AM0059/88 minigenome activity under heterologous polymerase conditions indicates that regulatory elements within the M-segment UTRs contribute to increased transcriptional or replicative efficiency independently of RdRp identity.

To explore potential molecular determinants underlying these differences, we performed sliding-window analysis of the RdRp amino acid sequence. The polymerase of OROV 2400023 and AM0059/88 displayed multiple regions of divergence relative to BeAn19991, particularly within the N-and C-terminal regions (Fig. 4d). While the N protein was conserved entirely across all three strains, these RdRp differences may contribute to enhanced polymerase processivity or stability. In addition, we noted three shared nucleotide differences in the M segment 3′ UTR of OROV 2400023 and AM0059/88 that were absent in BeAn19991 (Fig. 4e). The 5′ UTR was fully conserved across all strains, suggesting that these 3′ UTR polymorphisms could also influence replication or transcriptional efficiency through altered UTR secondary structures or interaction with the polymerase complex.

### Ancestral and contemporary OROV isolates exhibit comparable pathogenesis but altered neutralization profiles

**Fig. 5:**
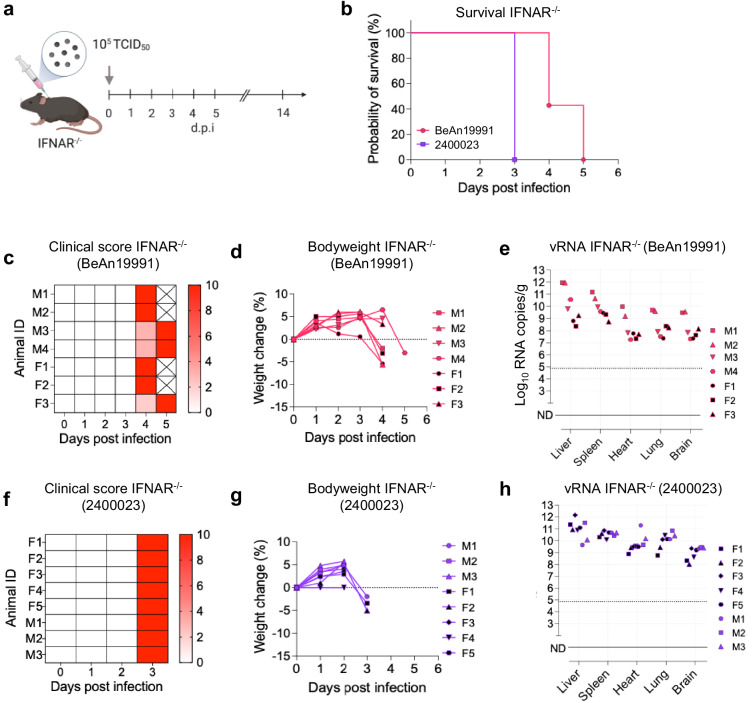
Pathogenesis of ancestral and contemporary OROV isolates in IFNAR^−/−^ mice. **a** Schematic of experimental design. (Created in BioRender. Tilston-lunel, N. (2026) https://BioRender.com/ ldb9voq). Six-week-old IFNAR^−/−^ male and female mice were infected SC with 10^5^ TCID_50_ of either rOROV BeAn19991 or OROV 2400023. **b** Kaplan–Meier survival curves for mice infected with rOROV BeAn19991 or OROV 2400023. Clinical disease scoring matrix for each animal infected with BeAn19991 (**c**) or 2400023 (**f**) with dpi shown on the x-axis and cumulative clinical scores indicated by heatmap scale (red = higher severity; X = euthanasia). Percent weight change over time in individual male (M) and female (F) mice infected with BeAn19991 (**d**) or 2400023 (**g**). vRNA loads in liver, spleen, heart, lung, and brain tissues of infected mice, measured by RT-qPCR at euthanasia. Data are shown for individual mice infected with BeAn19991 (**e**) or 2400023 (**h**). The dashed lines represent the limit of detection, ND not detected.

To evaluate the in vivo pathogenicity of contemporary OROV isolate 2400023, we used our validated lethal IFNAR^-/-^ mouse model^31^. IFNAR^−/−^ mice were inoculated with 10^5^ TCID_50_ of either rOROV BeAn19991 or OROV 2400023 and monitored for survival and disease progression (Fig. 5a). All animals infected with either strain succumbed to disease, with mortality occurring between 4 and 5 dpi for BeAn19991 and 3 dpi for 2400034 (Fig. 5b). Both groups exhibited a rapid onset of clinical disease, including ruffled fur, hunched posture, and weakness (Fig. 5c, e). In line with disease progression, animals infected with either strain exhibited an initial increase in body weight followed by a rapid decline prior to euthanasia or death, with losses of approximately 2 - 6% ^31^ (Fig. 5d, f). High vRNA loads were detected across all major organs, including liver, spleen, heart, lung, and brain, confirming widespread viral dissemination (Fig. 5e, g). In BeAn19991-infected animals, vRNA levels ranged from 8.4 to 11.9 and 8.7 to 11.2 log_10_ RNA copies/g in liver and spleen, respectively, and approximately 7–10 log_10_ RNA copies/g in heart, lung, and brain. In OROV 2400023-infected animals, vRNA levels ranged from 9.6 to 12.2 and 10.1 to 10.9 log_10_ RNA copies/g in liver and spleen, respectively, while vRNA levels in heart, lung, and brain were approximately 8–11 log_10_ RNA copies/g. Pale livers were observed at necropsy in several animals from both groups. Overall tissue viral loads and disease kinetics were comparable between strains. Despite this, seizure activity was observed in two animals infected with OROV 2400023, a clinical feature not previously noted in BeAn19991-infected mice under these experimental conditions.

**Fig. 6:**
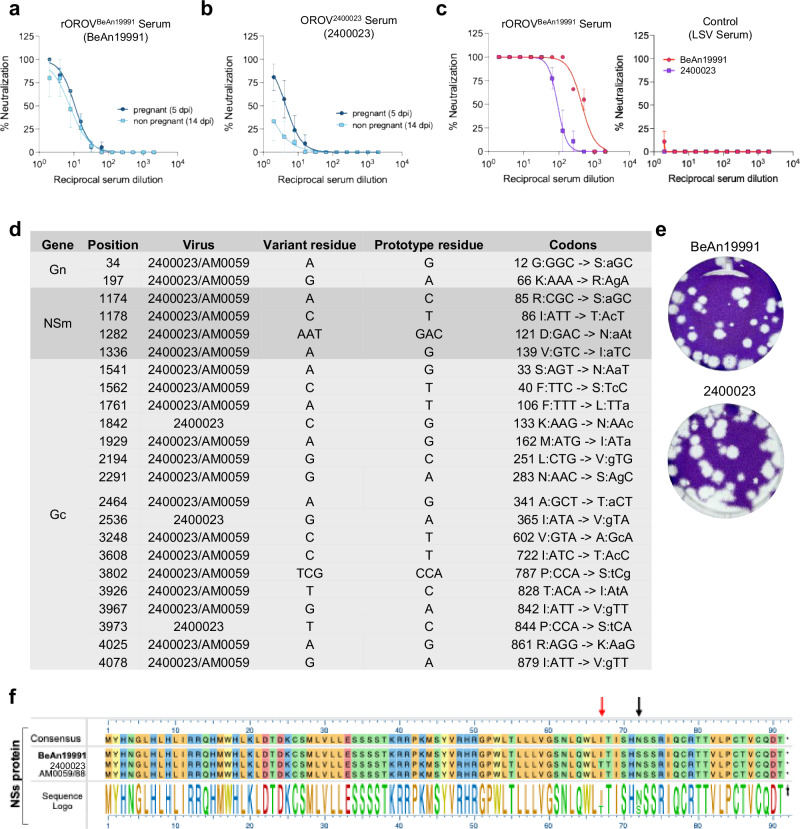
Contemporary OROV isolate 2400023 exhibits reduced neutralization sensitivity and defined sequence polymorphisms relative to the ancestral strain. Neutralization curves showing percent inhibition following incubation of virus with serially diluted sera from C57BL/6J mice infected at mid-gestation (E12.5; 5 dpi), or non-pregnant controls (14 dpi), infected with either (**a**) rOROV BeAn19991 [pregnant *n* = 7; non-pregnant *n* = 5; mean ± SE] or (**b**) OROV 2400023 [pregnant *n* = 6; non-pregnant *n* = 5; mean ± SE]. **c** Neutralization curves showing percent inhibition with serum from rOROV BeAn19991-infected C57BL/6J mice against both rOROV BeAn19991 (red) or OROV 2400023 (purple). Control sera from Lone Star virus (LSV)infected C57BL/6J mice were included to confirm assay specificity. (*n* = 3 biologically independent mice; mean ± SE). **d** Nonsynonymous coding changes in the M segment (Gn, NSm, Gc) of OROV 2400023 and/or AM0059/88 compared to BeAn19991. Codon substitutions and corresponding amino acid changes are indicated. **e** Plaque morphology of rOROV BeAn19991 (top) and OROV 2400023 (bottom) on Vero E6 cells at 3dpi visualized by crystal violet staining. **f** Amino acid alignment of the NSs from BeAn19991, 2400023, and AM0059/88. The sequence logo highlights conserved and variable residues. Black and red arrows denote polymorphisms of interest, including a unique substitution in 2400023 (red arrow).

Given the overall comparable in vivo pathogenicity observed between rOROV BeAn19991 and OROV 2400023 in the IFNAR^−/−^ model, we next examined whether differences in immune responses emerged in the C57BL/6J model. We therefore assessed virus-neutralizing activity in sera collected from C57BL/6J mice infected at mid-gestation (E12.5; 5 dpi) and included sera from non-pregnant, virus-infected mice collected at 14 dpi for comparison (Fig. 6a, b). Sera from BeAn19991-infected mice exhibited higher neutralizing activity overall compared with sera from OROV 2400023-infected mice, which showed markedly reduced neutralization at these early timepoints. This pattern was observed in both pregnant and non-pregnant cohorts (Fig. 6b). Notably, the number of mice with minimal or undetectable neutralizing activity was higher in the OROV 2400023 group than in the BeAn19991 group. These differences may explain the reduced maternal viral loads and, consequently, lower placental and fetal viral titers observed in this group compared with the BeAn19991-group (Figs. 1 and 2).

Next, to determine whether OROV 2400023 exhibits cross-neutralization properties similar to those reported for the contemporary Brazilian isolates AM0088 and AM0059^37^, we assessed the ability of serum from rOROV BeAn19991-infected C57BL/6J mice to neutralize OROV 2400023. Consistent with the phenotype described for the Brazilian isolates, sera from BeAn19991-infected mice showed reduced neutralizing activity against OROV 2400023 in comparison to rOROV BeAn19991 (Fig. 6c; Supplementary Fig. 7a). These findings confirm that OROV 2400023 shares a reduced cross-neutralization profile with other contemporary circulating OROV isolates across Brazil, despite comparable in vivo pathogenicity to the prototype.

Sequence comparison between OROV 2400023 and BeAn19991 revealed multiple nonsynonymous substitutions, including changes within the Gc glycoprotein that overlap regions implicated in antibody recognition in related orthobunyaviruses (Fig. 6d). While these substitutions provide a potential molecular basis for the observed cross-neutralization phenotype, the phenotypic consequences of these changes differ from those reported for AM0059^37^. OROV 2400023 did not exhibit a statistically significant difference in plaque morphology compared to rOROV BeAn19991 under our experimental conditions [unpaired t-test, Welch’s t-test, *t* = 1.87, df = 14.33, *p* = 0.0818; mean plaque diameters: 2.4 mm (BeAn19991) vs 2.7 mm for (2400023)] (Fig. 6e). Sequence alignment of the NSs protein, a known IFN antagonist, revealed differences among BeAn19991, 2400023, and AM0059, including conserved substitutions shared between OROV 2400023 and AM0059 as well as one unique variation present only in OROV 2400023 (Fig. 6f, red arrow). Although NSs are not a target of neutralizing antibodies, these differences may influence virus-host interactions that indirectly shape immune responses during pregnancy.

### Maternal infection with rOROV BeAn19991 confers partial protection to offspring through the production of neutralizing antibodies

**Fig. 7:**
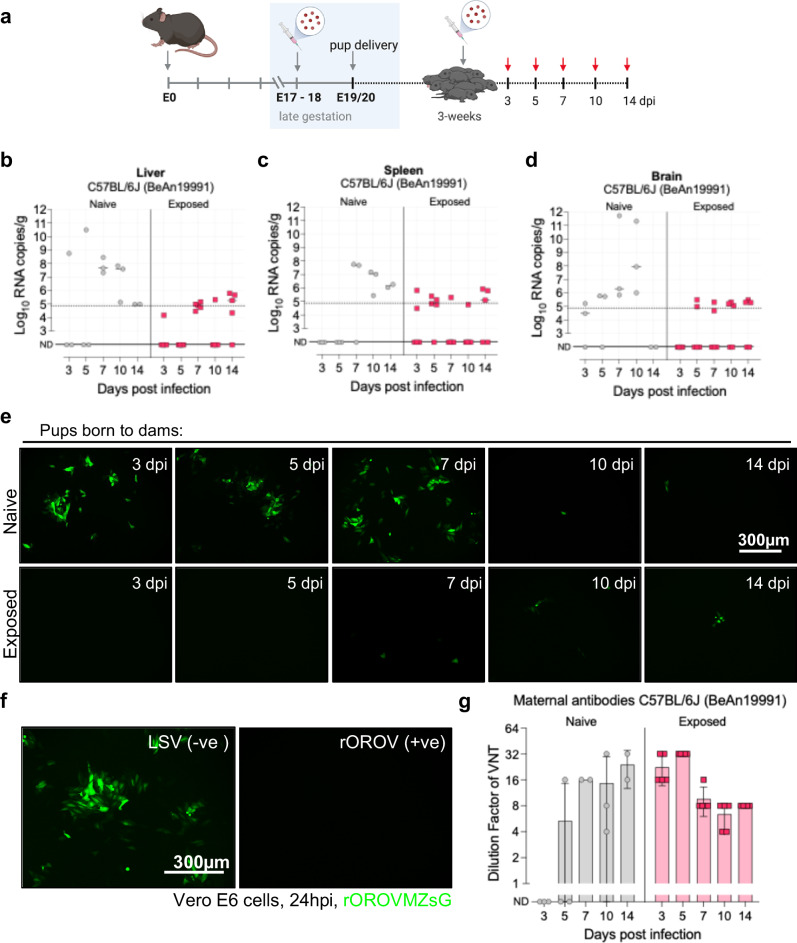
Maternal rOROV BeAn19991 infection confers partial protection to offspring via neutralizing antibodies. **a** Schematic of experimental design. (Created in BioRender. Tilston-lunel, N. (2026) https://BioRender.com/ ldb9voq). Viral load per gram of tissue in the liver (**b**), spleen (**c**), and brain (**d**) of pups, as measured by RT-qPCR. Each point represents an individual pup (infected dams: *n *= 5 pups per timepoint; uninfected dams: *n* = 3 pups per timepoint). Pups born to rOROV-exposed dams (red squares). Pups born to rOROV naive dams (gray circles). **e** Fluorescence images of rOROVMZsG-based virus neutralization test (VNT) in Vero E6 cells using serum collected from challenged pups. Fluorescence microscopy at 24 hpi using serum from pups born to uninfected or rOROV BeAn19991-exposed dams. ZsGreen signal (green) indicates infection in the presence of serum collected at 3, 5, 7, 10, and 14 dpi. Images are representative of independent pups with similar results (**f**) Representative images of rOROVMZsG-based VNT in Vero E6 cells using serum collected from LSV-infected (left, negative) and rOROV BeAn19991-infected (right, positive) controls at 24 hpi. **g** Quantification of neutralization titers from fluorescence readout. Each point represents serum from an individual pup (infected dams: *n* = 5 pups per timepoint; uninfected dams: *n* = 3 pups per timepoint), shown as mean ± SD. Dilution factors indicate peak neutralizing activity at early timepoints, with higher titers in pups born to exposed dams.

Lastly, given OROV’s ability to cross the maternal-fetal barrier, we investigated whether maternal infection could influence the susceptibility of offspring to subsequent infection. Specifically, we tested whether infection during pregnancy with the prototype strain rOROV BeAn19991 conferred protective immunity to pups following post-weaning challenge. To do this, we challenged pups born to mock-infected (naive) or rOROV-infected (exposed) C57BL/6J dams at 3 weeks of age and monitored infection outcome over 2 weeks (Fig. 7a). vRNA levels in the liver, spleen, and brain were measured at 3, 5, 7, 10, and 14 dpi. Pups born to naive dams exhibited high levels of vRNA across all tissues, with liver and spleen values reaching up to 8.5 and 7.8 log_10_ RNA copies/g at 7 and 10 dpi and brain vRNA values exceeding that in one animal at each time point (Fig. 7b–d). Notably, one pup succumbed to disease at 7 dpi and exhibited high brain vRNA levels over 11 log_10_ RNA copies/g. Additionally, pups displayed clinical signs of disease, including hunched posture and ruffled fur, at 10 dpi. In contrast, pups born to rOROV-exposed dams exhibited markedly reduced vRNA levels at all time points. In these animals, vRNA levels in liver, spleen, and brain were consistently near or below the LOD, rarely exceeding approximately 4.5–5.5 log_10_ RNA copies/g, and no clinical symptoms were observed. These findings indicate substantial, though incomplete, protection against systemic viral replication in offspring born to previously infected dams.

To directly assess the contribution of neutralizing antibodies, we employed a fluorescence-based virus-neutralization assay using our rOROVMZsG reporter virus^31^. This assay enabled rapid visualization of inhibition by monitoring ZsGreen expression in infected cells. Sera from pups born to rOROV-exposed dams exhibited strong, dose-dependent inhibition of reporter virus at early time points (3–7 dpi). In contrast, sera from pups born to naive dams showed little to no inhibition until 10 dpi (Fig. 7e, f). Quantification of end-point neutralization titers revealed peak antibody activity in pups from exposed dams between 3 and 7 dpi, followed by a decline by 10–14 dpi, consistent with transient, maternally derived protection (Fig. 7g; Exposed, red). To confirm these findings, we performed a complementary crystal violet-based assay (Supplementary Fig. 7b), which produced concordant results. Collectively, these data show that maternal OROV infection generates strong but transient neutralizing antibody-mediated responses in offspring, resulting in partial protection from post-weaning challenge.

## Discussion

Congenital infection and fetal disease are well-documented features of several related orthobunyaviruses, including Schmallenberg virus (SBV)^38^, Akabane virus (AKAV)^39^, Cache Valley virus^40^, and Shuni virus^41^, where vertical transmission frequently results in placental infection, fetal malformations, central nervous system (CNS) involvement, and, in some cases, long-term viral persistence in ruminant hosts. These viruses establish a clear precedent for transplacental transmission within the *Orthobunyavirus* genus. Despite this, OROV has historically been associated with self-limiting febrile illness, and its capacity to impact pregnancy has remained poorly defined. The 2022 – 2024 outbreak highlighted its association with adverse pregnancy outcomes, including suspected maternal-to-fetal transmission^20,21^, motivating direct experimental investigation. Accordingly, we combined in vivo and in vitro approaches to (a) establish a murine pregnancy model for OROV infection and (b) compare a contemporary outbreak-associated isolate (OROV 2400023, Cuban 2024) with the ancestral prototype strain (rOROV BeAn19991, Brazil 1960). Using timed gestational infections in immunocompetent C57BL/6J mice, we demonstrate that placental infection is a conserved feature of OROV biology and not a newly acquired feature of contemporary strains.

Although vertical transmission potential was conserved, the contemporary OROV 2400023 isolate showed clear evidence of enhanced replicative fitness, consistent with our previous observations in midge-derived cells^14^. OROV 2400023 replicated faster than BeAn19991 in both IFN-competent and IFN-deficient cell lines, particularly during early infection. It is plausible that this accelerated replication kinetics also contributed to the higher frequency of early gestation placental infection observed in vivo compared to the ancestral strain. To investigate potential molecular determinants underlying these differences, we performed minigenome assays using a contemporary Brazilian isolate (AM0059/88). Here, AM0059/88 consistently outperformed BeAn19991 in reporter activity. While this enhancement is likely influenced by variation in the RdRp, we also identified mutations in the 3′ UTR of the M segment that may affect cis- or trans-acting regulating elements. As we have previously shown, mutations in OROV UTRs can strongly influence minigenome activity^42^. Ongoing studies are focused on dissecting how these sequence changes impact viral gene expression, replication efficiency, and virulence.

Scachetti et al. ^37^ previously reported that human and murine sera raised against BeAn19991 poorly neutralized OROV AM0059, indicating reduced cross-neutralization between ancestral and contemporary strains. While our neutralization assays were not epitope-resolved and do not support a claim of antigenic divergence, we did observe reduced neutralization efficiency of OROV 2400023 by sera raised against BeAn19991. Given that OROV 2400023 and AM0059/88 share multiple conserved substitutions within Gc, these strains may exhibit similar antigenic features. These findings highlight that sequence variation within Gc can influence neutralization sensitivity, raising the possibility that prior exposure to circulating strains may provide incomplete protection against emerging OROV variants. Supporting this interpretation, prior work using vesicular stomatitis virus (VSV)-based OROV vaccine chimeras identified the N-terminal head domain of Gc as immunodominant but dispensable for protection^43^. Deletion of this variable domain redirected antibody response toward more conserved regions and improved protective efficacy, suggesting that natural infection may preferentially elicit strain-specific immunity that limits cross-protection. Our sequence analysis confirms that contemporary OROV strains harbor substitutions within this same region of Gc. Studies of related orthobunyaviruses, including Bunyamwera virus (BUNV), SBV, and AKAV, have shown that neutralizing antibody responses are primarily directed against the Gc ectodomain^44–46^. Future studies using monoclonal antibodies and targeted reverse-genetics approaches will be required to map strain-specific Gc epitopes and define their contribution to immune evasion.

In addition to structural protein variation, changes in non-structural proteins NSm and NSs may also influence fitness and host interactions. We have previously shown that NSm is dispensable for OROV in the mammalian host^31,32^, but its role in vector competence remains unresolved. Several variant residues are shared between 2400023 and AM0059/88 and differ from BeAn19991, including substitutions that may affect NSm function. Investigating the phenotypic consequences of these mutations will help determine whether NSm contributes to vector-specific replication or transmission, as observed for other orthobunyaviruses such as BUNV^47^. Within the NSs, both 2400023 and AM0059/88 encode an asparagine residue at position 71, while 2400023 encodes a threonine residue at position 67 uniquely. We have previously demonstrated that deletion of NSs results in robust type I IFN production, STAT1 phosphorylation, and upregulation of antiviral effectors such as MxA^32^. Because NSs overlaps the N gene in an alternative reading frame, such changes likely reflect evolutionary pressure to maintain N function while fine-tuning host immune antagonism. Whether these substitutions enhance NSs’ stability, IFN suppression, or host-specific interactions is currently under investigation.

While these molecular features shape viral replication and immune evasion, our study also uncovered important immunological consequences of OROV infection during pregnancy. We found that maternal OROV infection elicited neutralizing antibodies that are transferred to offspring, conferring partial protection upon homologous challenge. Given the timing of infection and delivery, this protection is most consistent with postnatal antibody transfer, likely mediated through milk, rather than in utero fetal immunization^48,49^. These findings parallel observations from other vertically transmitted arboviruses, including Zika^50,51^ and dengue virus^52^, and highlight the potential utility of maternal immunization strategies for protecting neonates in OROV-endemic regions.

Although several orthobunyaviruses exhibit marked neurotropism, including OROV^53,54^, we observed limited fetal infection following gestational exposure in immunocompetent dams. Across both ancestral and contemporary strains, placental tissues consistently harbored higher vRNA loads than matched fetal tissues, indicating that while OROV efficiently replicates at the maternal-fetal interface, fetal dissemination is comparatively constrained in this model. The importance of host type I IFN signaling was underscored by experiments in our IFNAR^-/-^ x WT cross, which served as a proof-of-principle model for congenital infection. In this setting, placental infection was uniformly high, widespread fetal dissemination was readily detected, and infectious virus was recovered from fetal tissues, despite the absence of gross fetal malformations. Rather than acting as a direct barrier at the maternal-fetal interface, our data suggest that reduced type I IFN signaling in the dams promotes systemic viral replication, thereby increasing the likelihood of placental infection and subsequent viral dissemination. These findings are consistent with prior studies^55^, including our previous work^31^ demonstrating that loss of type I IFN signaling permits uncontrolled OROV replication, systemic dissemination, and severe disease. In line with this, both ancestral and contemporary OROV strains in this study produced rapid, lethal infection in IFNAR^−/−^ mice, with high viral burdens across multiple organs. Together, these data reinforce the central role of type I IFN signaling in restricting OROV pathogenesis. It is important to note that species-specific differences in innate immune responses may limit the extent to which these findings reflect human infection.

Beyond innate immune differences, additional limitations of the murine system should be considered when interpreting these findings. Murine gestation (~19–21 days) differs substantially from human pregnancy in both duration and developmental timing, particularly with respect to placentation and fetal organogenesis. Although mice and humans share a hemochorial placental architecture, the timing of placental maturation, trophoblast invasion, and maternal-fetal immune interactions is not directly comparable. In addition, comparative studies of related orthobunyaviruses, including SBV and AKV, indicate that congenital disease phenotypes observed in natural ruminant hosts, such as fetal malformations and neurotropism, are only partially recapitulated in murine systems^39,56–59^. In addition, infections in this study were performed under controlled conditions using direct inoculation rather than natural vector-mediated transmission, which may alter viral dose, kinetics, and tissue tropism relative to natural exposure. Together, these considerations frame the murine pregnancy model as an experimentally accessible but inherently limited system for studying congenital OROV infection, warranting cautious extrapolation to human pregnancy.

In conclusion, our study establishes vertical transmission as a conserved feature of OROV biology and identifies the placenta as a primary site of viral replication during pregnancy. By integrating immunocompetent and IFN-deficient pregnancy models, we define a framework in which placental infection is readily supported, whereas fetal dissemination is modulated by host immune competence and developmental context. This work reframes OROV as a virus of relevance to maternal-fetal health and provides a mechanistic basis for understanding how host immunity shapes congenital risk during OROV infection.

## Methods

### Cells, viruses, and plasmids

Vero E6 cells (African green monkey kidney cells), A549 (human alveolar adenocarcinoma epithelial cells) and BSR-T7/5 cells^60^ (baby hamster kidney cells), which stably express T7 RNA polymerase were grown in Dulbecco’s modified Eagle medium (DMEM; Gibco) supplemented with 2–10% (V/V) fetal bovine serum (FBS; Gibco). BeWo cells (choriocarcinoma-derived trophoblasts) were grown in Kaighn’s Modification of Ham’s F-12 Medium (F-12K; ATCC) supplemented with 10% (V/V) FBS. JEG3 cells (choriocarcinoma-derived trophoblasts) were grown in Eagle’s minimal essential medium (EMEM; Sigma Aldrich) supplemented with 10% (V/V) FBS. HTR8 cells (HTR8/SVneo first-trimester transformed cells) were grown in RPMI-1640 supplemented with 10 mM HEPES, 1 mM sodium pyruvate, and 10% (V/V) FBS. All cells were grown at 37 °C and 5% CO_2_.

We have previously described^31,32^ both rOROV^BeAn19991^ and rOROVMZsG. OROV^2400023^ was obtained from the Arbovirus Reference Collection at the University of Texas Medical Branch (UTMB). For simplicity, throughout the manuscript and figures, rOROV^BeAn19991^ and OROV^2400023^ are referred to by their strain designations, such as rOROV ancestral strain BeAn19991 and OROV strain 2400023, respectively. OROV 2400023 was passaged 3 times in Vero E6 cells at UTMB and once upon arrival at our facility. To obtain pure stocks, we plaque-purified 2400023. Here, infected Vero E6 cells were overlaid with 0.6% low-melt agarose (Fisher Scientific, BP16525) in 2× minimum essential medium (MEM) with 2% FBS. 4 dpi cells were stained with 0.33% Neutral Red solution (Sigma-Aldrich) to visualize plaques. Plaques were then picked using a pipette tip to infect Vero E6 cells (12-well plate, 2 × 10^5^ cells/ml). 48 hpi infectious virus supernatants were harvested. Virus stocks were grown and titrated in Vero E6 cells using a 50% Tissue Culture Infectious Dose Assay (TCID_50_). Virus titers for rOROV BeAn19991, rOROVMZsG, and OROV 2400023 were 2.42 × 10^6^ TCID_50_/ml, 2.7 × 10^5^ TCID_50_/ml, and 1.1 × 10^7^ TCID_50_/ml, respectively. All virus stocks used in this study were low-passage, sequence-verified, and matched across experiments where direct comparisons were made.

The OROV BeAn19991 minigenome and expression plasmids (pT7-BeAn19991-M:hRen, pTM1-N, pTM1-RdRp, pTM1-FFLuc, and pTM1-empty vector) have been previously described^42^. The contemporary M-segment minigenome reporter plasmid (pT7-AM0059/88-M:hRen) was generated by cloning the AM0059/88 M-segment UTRs into a pT7 backbone containing a humanized *Renilla* luciferase reporter (hRen). The coding sequences of the AM0059/88N and RdRp proteins were cloned into the pTM1 backbone. All constructs were assembled using an In-Fusion® enzyme-based restriction-free cloning strategy^42^.

### RT-PCR and Sanger sequencing of OROV 2400023

vRNA from OROV 2400023 was extracted from 250 μl of viral supernatant using 750 μl of TRIzol LS Reagent (Ambion) and purified with the Direct-zol RNA MiniPrep kit (Zymo Research), following the manufacturer’s instructions. Complementary DNA (cDNA) synthesis was performed using M-MuLV reverse transcriptase (New England Biolabs). Segment-specific primers were used for PCR amplification of each genome segment: S segment (priOROVSF-FL: AGTAGTGTACTCCACAAT and priOROVSR-FL: AGTAGTGTGCTCCCAATT), M segment *fragment 1* (priOROV-MF-new-FL: AGTAGTGTACTACCAGCAACAA and priT7AM0059M4045R: GCAAAGCAGGTGATTTGTGTGC), M segment *fragment 2:* (priT7AM0059M2186F: GTAGATCCACCCGCTCAG and priOROV-MR-new-FL: AGTAGTGTGCTACCAACA), L segment *fragment 1* (priT7AM00590L91F: ATGTCGCAACTGTTACTC and priT7AM00590L4148R: GCTTAACAGGTATCTCTG), L segment *fragment 2* (priT7AM00590L2213F: CAGACTACGATATAACAC) and priT7AM00590L6328R: CTTATCATGTATGCTAGTG) and L segment *fragment 3* (priT7AM00590L3623F: CATCCATATGTATGGTGC and priT7AM00590L8546R: CTTAGAAGTCAAATTTGG). PCR amplification of the S segment was performed using GoTaq™ G2 Master Mix (Promega) in a thermal cycler (Applied Biosystems) with touchdown PCR (annealing temperatures: 45 °C, 48 °C, and 50 °C). M segment cDNA was amplified under standard PCR conditions with an annealing temperature of 56 °C. PCR products were resolved by agarose gel electrophoresis, and bands of the expected size were excised and purified using a gel extraction kit (IBI Scientific). Purified DNA was submitted for Sanger sequencing and analyzed using SeqMan Ultra, DNASTAR (V18.0.3.2).

### Viral growth kinetics

Vero E6 and A549 cells (2 × 10^5^ cells/ml, 24-well plate) were infected with either rOROV BeAn19991 or OROV 2400023 at an MOI 0.1 for 1 h at 37 °C. Cell monolayers were then washed 3× with D-PBS (Gibco) and then provided with a 2% FBS growth medium. At desired time points, supernatants were collected and harvested, and infectious viral titers were determined by a TCID_50_ assay. HTR8, BeWo, and JEG3 cells (1 × 10^5^ cells/ml, 24-well plate) were infected with either rOROV BeAn19991 or OROV 2400023 at an MOI of 0.002 for 1 h at 37 °C. Cell monolayers were then washed 3× with D-PBS (Gibco) and then provided with a 10% FBS growth medium. At desired time points, supernatants were collected and harvested, and a TCID_50_ assay was used to determine infectious viral titers.

### TCID_50_ and plaque assays

TCID_50_ assays were performed in Vero E6 cells and seeded at a density of 10^4^ cells per well in 96-well plates and infected with a 10-fold serial dilution of virus. At 5–7 dpi, CPE was recorded, and viral titers were expressed as tissue culture infectious dose (TCID_50_ units). Plaque assays were performed in 6-well plates with Vero E6 cells at a density of 10^5^ cells/ml. Cell monolayers were infected with a 200 µl inoculum of virus diluted in Opti-MEM. Cells were then overlaid with 0.6% Avicel (FMC, Avicel RC-591) in 2× minimum essential medium (MEM)/2% fetal bovine serum (FBS). Cells were fixed at 3–4 dpi with 4% (wt/vol) paraformaldehyde (PFA) in phosphate-buffered saline (PBS) for 30 min, and plaques were visualized using crystal violet.

### Minigenome assays

Sub-confluent BSR-T7/5 cell monolayers were seeded in 24-well plates at 1.4 × 10^5^ cells/ml and co-transfected with 500 ng of pT7-BeAn19991-M:hRen or pT7-AM0059/88-M:hRen reporter plasmid. For N protein titration experiments, cells were co-transfected with increasing amounts of pTM1-N (0–1000 ng) and constant amounts of pTM1-RdRp (250 ng). For RdRp titration experiments, cells received increasing amounts of pTM1-RdRp (0–1000 ng) with constant amounts of pTM1-N (250 ng). pTM1-FFLuc (Firefly luciferase; 5 ng) served as an internal normalization control, and pTM1-empty vector was added to maintain equal total DNA amounts across all transfections.

Transfection complexes were prepared by combining plasmid mixtures with 25 μl Opti-MEM (ThermoFisher Scientific) containing 1 μl P3000 enhancer reagent. Separately, lipid transfection mixtures were prepared in 25 μl Opti-MEM containing Lipofectamine 3000 reagent (0.6 μl per μg total DNA; ThermoFisher Scientific). After incubation and complex formation according to manufacturer’s protocol, transfection mixtures were added to cell monolayers. Cells were lysed 24 h post-transfection (hpt), and *Renilla* and firefly luciferase activities were measured using the Dual-Luciferase Reporter Assay System (Promega) according to manufacturer’s instructions. *Renilla* luciferase values were normalized to firefly luciferase activity to account for transfection efficiency variations.

### Immunofluorescence

Confluent monolayers of Vero E6, HTR8, BeWo, or JEG3 cells were infected with either rOROV BeAn19991 or OROV 2400023 at a set volume for virus isolation or an MOI of 0.1. After 24 h of incubation at 37 °C, 5% CO_2_, the supernatant was discarded, and the cell monolayer was washed with phosphate-buffered saline (PBS) and fixed using 4% (w/v) paraformaldehyde in PBS. After 20 min of incubation at room temperature (RT), the fixative was removed, and the monolayer was washed twice with PBS. Cell monolayers were then permeabilized (0.5 % Triton X-100, 20 mM sucrose in PBS; 1 ml) for 30 min at RT. Cells were then incubated with 200 µl mouse anti-OROV diluted 1:500 in 1 % Bovine Serum Albumin (BSA)/0.1 % Tween-20 in PBS for 1 h at RT. Cells were washed several times before incubating with 200 µl Alexa Fluor 594 goat anti-mouse IgG (H&L) diluted 1: 1000 in 1 % BSA/0.1 % Tween-20 in PBS for 1 h at RT. After washing the cells three times with PBS, 4′,6-diamidino-2phenylindole (DAPI) nuclear stain (Fisher EN2248) was added and incubated in the dark for 10 min at RT. BeWo cells were incubated with 200 µl Alexa Fluor 488 phalloidin (Invitrogen A12379) diluted 1:500 in PBS for 15 min at RT to stain cellular actin. Images were obtained using a fluorescence microscope (EVOS M5000 imaging system). Phalloidin staining was included only for BeWo cells to confirm that the cells were not syncytialised during infection. This control was not required for the other cell lines, which do not undergo syncytialisation.

### Animal study design

(a) Pathogenesis study in C57BL/6J mice: Six-week-old female C57BL/6J mice (Strain #000664, Jackson Laboratory; IUSM colony) were housed under specific pathogen-free conditions in HEPA-filtered cages within an ABSL-2 facility, with ad libitum access to food and water. Mice were subcutaneously (SC) inoculated with 10^5^ TCID_50_ of rOROVMZsG diluted in Opti-MEM to a final volume of 100 μl. Control animals received UV-inactivated rOROVMZsG (Benchmark UV Clave chamber; 6 × 4-W UV bulbs; 150 mJ/cm^2^) at the same dose and volume. Mice were monitored daily for clinical signs, and body weights were recorded. On 5, 7, and 14 dpi, animals were anesthetized with isoflurane and terminally exsanguinated for serum collection, followed by euthanasia via cervical dislocation. Each time point *n* = 3. Tissues, including liver, spleen, heart, lungs, and brain, were collected for viral isolation and RNA extraction. For viral load analysis, organs were placed in pre-weighed tubes containing 500 μl PBS supplemented with 2× Antibiotic-Antimycotic (Gibco), weighed, and homogenized using a Bead Mill 24 Homogenizer (Fisherbrand). A 100 μl aliquot of homogenate was mixed with 400 μl TRIzol LS reagent (Ambion) and processed using the Direct-zol RNA Purification Kit (Zymo Research). (b) Timed pregnancy studies: Six-week-old female C57BL/6J or IFNAR^−/−^ mice (Jackson Laboratory, IUSM colony) were time-mated with male C57BL/6J mice and housed under the conditions described above. The presence of a vaginal plug confirmed pregnancy, and at the indicated embryonic days, pregnant dams were subcutaneously inoculated with OROV. For the WT C57BL/6J experiments dams were infected with 2.42 × 10^5^ TCID_50_ of rOROV BeAn19991 or OROV 2400023. The IFNAR^−/−^ dams were infected with 10^2^ TCID_50_ rOROV BeAn19991. Mice were monitored daily and weighed until euthanasia. Dams were anesthetized with isoflurane and terminally exsanguinated, followed by cervical dislocation. Maternal tissues (liver, spleen), placentas, and pups were collected for viral isolation and RT-qPCR analysis. Pups were euthanized by decapitation under ice water anesthesia or fixed in 10% buffered formalin (Fisher) for histological analysis. Maternal organs were processed as above using 500 μl PBS with 4x Antibiotic-Antimycotic. For pup viral loads, whole pups were collected in pre-weighed tubes containing 3 ml PBS with 4× Antibiotic-Antimycotic, weighed, homogenized, and 250 μl of homogenate was mixed with 750 μl TRIzol LS for RNA extraction. (c) Pathogenesis study in IFNAR^−/−^ mice: Six-week-old male and female IFNAR^−/−^ mice (B6(Cg)-*Ifnar1*^*tm1.2Ees*^*/J*, strain #: 028288, Jackson Laboratory; IUSM colony) were housed in the ABSL-2 facility as described above. Mice were SC infected with 10^5^ TCID_50_ of either rOROV BeAn19991 (*n* = 7; 4 males and 3 females) or OROV 2400023 (*n* = 8; 3 males and 5 females) diluted in Opti-MEM to a final volume of 100 μl. At euthanasia, mice were anesthetized and terminally exsanguinated. Tissues (liver, spleen, heart, lungs, brain) were collected for RT-qPCR and fluorescence analysis using an EVOS M5000 imaging system (ThermoFisher). For histology, tissues were fixed in 4% PFA, paraffin-embedded, and sectioned (5 μm) for hematoxylin and eosin (H&E) staining by the Indiana CTSI Histology Core.

### RT-qPCR assay

vRNA was quantified targeting the S genome segment^31^ using the following primers and probes: rOROV BeAn19991: rOROV-S qPCR primers (Fwd: GCGTCACCATCATTCCAAGTA, Rev: CCCAGATGCGATCACCAATTA) and OROV-SqPCRprobe (FAM: AGCCACTGTAGTAGTGCCTTTGG), and OROV 2400023: OROV-TVP-S qPCR primers (Fwd: TACATCGCGTCACCATCATTC, Rev: CCCAGATGCGATCACCTATTAAG) and OROV-TVP-SqPCRprobe (FAM: TTGGCAGAGGTGAAGGGTTGTACT). The working concentration for the primers was 10 μM for the probe 5 μM. To generate an OROV 2400023 S RNA standard curve, an IDT gene fragment containing the sequenced OROV 2400023 S segment was synthesized and used as a template for in vitro RNA transcription (MEGAscript T7 transcription kit; Invitrogen). The resulting RNA was diluted to known copies per ml in RNase-free water and serially diluted for each assay. RT-qPCR was performed using the Luna Universal Probe One-Step RT-qPCR Kit (New England Biolabs). The assay was performed on the QuantStudio^™^ 5 (ThermoFisher Scientific) using the following conditions: 55 °C for 10 min, 95 °C for 1 min, and then 40 cycles of 95 °C for 10 s and 60 °C for 1 min. For the tissue samples, RNA copies were normalized to grams of tissue to allow comparison across organs of different sizes. RNA levels were reported as log vRNA copies per gram of tissue, with the limit of detection calculated based on the highest cycle threshold (CT) value detected in the standard curve for each assay.

### Hybridization chain reaction (HCR) for OROV vRNA detection in tissue sections

Probe sets targeting rOROV BeAn19991 S segment were designed using the DIY probe generator pipeline^61^. All probes were screened for specificity against host cell transcripts to minimize background. The HCR assay was performed following protocols published by Molecular Instruments, Inc. To visualize OROV vRNA, tissue sections were deparaffinized in Histoclear (3×5 min), followed by two washes in 100% ethanol (3 min each) to remove residual Histoclear. Sections were rehydrated using a graded ethanol series (95%, 85%, 70%, and 50% ethanol, each for 3 min) and rinsed in Milli-Q water for 3 min. For antigen retrieval, slides were immersed in 250 ml of 1× antigen unmasking solution (prepared from a 100× stock in Milli-Q water) within a plastic container, ensuring complete submersion. The container was placed in a pressure cooker and heated at maximum pressure for 20 min. After retrieval, slides were cooled to RT and incubated in 1x PBST (2 × 2 min). Tissue sections were then treated with 10 µg/ml proteinase K in PBST for 10 min at 37 °C in a humidified chamber, followed by two washes in fresh 1× PBST. Probe hybridization buffer (50–100 µl per section) was applied, and slides were incubated at 37 °C for 10 min in a humidified chamber. Hybridization was performed by adding a probe solution (0.4 pmol of each probe set; Supplemental Table; in 100 µL of hybridization buffer), followed by overnight incubation (>12 h) at 37 °C. The next day, slides were washed sequentially in probe wash buffer at 37 °C for 15 min, followed by graded washes in 75%, 50%, and 25% probe wash buffer diluted in 5× SSCT, and finally 100% 5x SSCT (15 min per step). Slides were then immersed in 5x SSCT at RT for 5 min before air-drying. For amplification, sections were incubated with an amplification buffer at RT for 30 min. Hairpin reagents (B3-647; Molecular Instruments, Inc.) were prepared by snap cooling 6 pmol of hairpins h1 and h2 separately (2 µl of 3 µM stock, heated at 95 °C for 90 s, then cooled to RT for 30 min in a dark tube). The snap-cooled hairpins were then mixed in 100 µl of amplification buffer. The pre-amplification solution was removed, and 50–100 µl of the hairpin solution was applied to each section, followed by overnight incubation (>12 h) in a dark, humidified chamber at RT. On the final day, slides were washed four times for 5 min each in 5x SSCT at RT. Nuclei were stained with 100 µl of 1 µg/ml Hoechst for 5 min, followed by a final wash in 5x SSCT. Sections were immediately mounted and visualized on the EVOS M5000 imaging system (ThermoFisher).

### Virus neutralization assays

(a) High-throughput assay using reporter virus: Mouse sera was heat-inactivated at 56 °C for 30 min in a thermal cycler (Applied Biosystems), then 2-fold serially diluted in FluoroBrite™ DMEM (Gibco) in 96-well dilution plates (CellTreat; 25 μl/well). Diluted rOROVMZsG (MOI 0.1) was added to each well and incubated at 37 °C for 1 h. Virus–serum mixtures were transferred to Vero E6 cells (10^4^ cells/well) seeded in black-walled 96-well plates (Corning). Sera from a Lone Star virus (LSV)-infected mouse served as a negative control. Plates were incubated for 48 h and read using a BioTek Synergy H1 plate reader. Whole-plate fluorescent images were acquired using an Odyssey M Imager (LI-COR). (b) Neutralization of ancestral and contemporary strains: Sera from rOROV BeAn19991-infected mice were heat-inactivated and serially diluted 2-fold in Opti-MEM (25 μl/well). Diluted rOROV BeAn19991 or OROV 2400023 (MOI 0.1) was added and incubated for 1 h at 37 °C before transfer to Vero E6 cells (10^4^ cells/well in 96-well plates). At 5 dpi, cells were fixed in 4% PFA (15 min) and stained with crystal violet. Neutralization titers were either defined as the highest serum dilution exhibiting <50% CPE or visually scored using a semi-quantitative scale as follows: no detectable CPE (−), mild CPE (+), moderate CPE (++), or extensive CPE (+++). CPE scores were converted to normalized percent neutralization values (− = 100%, + = 66%, ++ = 33%, +++ = 0%) to enable quantitative comparison across serum dilutions. Neutralization curves were generated by fitting normalized percent neutralization values using a variable-slope nonlinear regression model to visualize trends in CPE-based neutralization data.

### Data and statistical analysis

RT-qPCR data were processed in Microsoft Excel and visualized using GraphPad Prism (V10). Χ² analysis was used to compare rates of placental infection between strains. Mann-Whitney test was used to compare vRNA levels of placental infection between strains (Supplemental Fig. 4). Growth comparison of rOROV BeAn19991 and OROV 2400023 in human placental cell lines was analyzed by multiple unpaired t-tests with individual variance for each row, false discovery rate (FDR), and correction applied using the two-stage step-up method of Benjamini, Krieger, and Yekutieli in GraphPad Prism V10 (Fig. 3a–c). Growth comparison of rOROV BeAn19991 and OROV 2400023 in A549 and Vero E6 cells was analyzed by a Two-way ANOVA followed by Sidak’s multiple comparisons in GraphPad Prism V10 (Fig. 4). Sliding window analysis and visualization were performed in R (V4.4.0) and RStudio (V2024.04.1 + 682) (Fig. 4d). Sequencing alignments and visualization were performed using DNAstar (V18.0.3.2) (Fig. 6d, f).

### Ethics statement

All animal work was performed in compliance with Indiana University School of Medicine’s Institutional Animal Care and Use Committee (IACUC; protocol 22080; PI: Tilston) and Institutional Biosafety Committee (IBC). Experiments were performed in an Animal Biosafety Level 2 (ABSL-2) facility where animals were housed in an insect-secure environment with no vectors present, using microisolator cages and facility safeguards (double-door access, sealed windows, air curtains, and restricted entry) to prevent arthropod exposure and escape. Animals were monitored twice daily and euthanized upon reaching predefined humane endpoints^31^.

### Reporting summary

Further information on research design is available in the Nature Portfolio Reporting Summary linked to this article.

## Supplementary information

Supplementary Information

Peer Review file

Reporting Summary

## Source data

Source Data

## Data Availability

This study used publicly available sequences from GenBank, including contemporary OROV isolates from Brazil with the accession numbers: PP992525 (AM0088; S segment), PP992526 (AM0059; S segment), PP992527 (AM0088; M segment), PP992528 (AM0059; M segment), PP992529 (AM0088; L segment), and PP992530 (AM0059; L segment) as well as the prototype OROV strain BeAn19991: KP052852 (S segment), KP052851 (M segment) and KP052850 (L segment). Source data are provided with this paper. Additional information is available from the corresponding author (N.L.T.) upon reasonable request. Source data are provided with this paper.
