# Supplementary material for: Conserved pathogenesis of ancestral and contemporary Oropouche virus strains in a murine pregnancy model
Source: Nat Commun. 2026 May 12;17:6542. doi: 10.1038/s41467-026-72711-2 (PMC13381898; doi:10.1038/s41467-026-72711-2)
Supplement: Supplementary file 1 — Supplementary Information [file 41467_2026_72711_MOESM1_ESM.pdf]

SUPPLEMENTARY INFORMATION

Conserved pathogenesis of ancestral and contemporary Oropouche virus strains in a murine pregnancy model

Krista B. Gunter[1†], James M. Bowen[1†], Andrew T. Clarke[2], Melanie McFarlane[2], Dorcus C. A. Omoga[1], Stephanie Pozuelos[1], Henry Giesel[1], Curtis Witt[1], Lisa M. Rogers[3], David M. Aronoff[3], Andrew M. Lunel[4], Jay Vornhagen[1], Benjamin Brennan[2] and Natasha L. Tilston[1*]

[1]Department of Microbiology and Immunology, Indiana University School of Medicine, Indianapolis, Indiana, United States of America.
[2]MRC-University of Glasgow Centre for Virus Research, Glasgow, United Kingdom.
[3]Department of Medicine, Indiana University School of Medicine, Indianapolis, Indiana, United States of America.
[4]Department of Pediatrics, Herman B Wells Center for Pediatric Research, Indiana University School of Medicine, Indianapolis, Indiana, United States of America.

[†]Equal contribution
*Corresponding author: Natasha L. Tilston (Tilston-Lunel); ntilston@iu.edu

This PDF includes:
Supplementary Table
Supplementary Figures 1 to 7

Supplement Table: HCR probe pairs used in this study.

Supplemental Figure 1. rOROVMZsG in immunocompetent C57BL/6J mice. (a) Schematic of experimental design. (Created in BioRender. Tilston-lunel, N. (2026) https://BioRender.com/ldb9voq). WT C57BL/6J mice were infected SC with either rOROVMZsG (n=3 mice per group per timepoint) or UV-inactivated rOROVMZsG (n=3), and euthanized at 5, 7, or 14 dpi. (b) Percent weight change of mice from baseline compared to UV-inactivated virus controls. Data are shown as mean ± SD. (c) vRNA loads per gram of tissue in the liver, spleen, heart, lung, and brain of rOROVMZsG or UV-inactivated infected mice as measured by RT-qPCR. The dashed line represents the limit of detection, ND = not detected. (d) Representative fluorescence images from virus isolations in Vero E6 cells following inoculation with homogenized liver and spleen samples from infected mice (5 dpi), demonstrating recovery of infectious rOROVMZsG. UV-inactivated controls are shown for comparison. Scale bars as indicated. (EVOS M5000 imaging system, ThermoFisher).

Supplemental Figure 2. Infection with rOROV BeAn19991 causes liver pathology and placental infection. (a) Representative images of fetuses harvested from early or mid-gestation infected dams, showing normal gross morphology. (b) Detection of replication-competent virus in Vero E6 cells infected with placental homogenates. Immunofluorescence staining shows rOROV (red) at 24 hpi. Nuclei stained with DAPI (blue). Placental isolates from three difference dams are shown. (c) Sole positive detection of replication-competent virus in Vero E6 cells infected with fetal homogenate (D1, F4). (c) Representative H&E-stained liver sections from mock- and rOROV-infected pregnant C57BL/6J dams.

Supplemental Figure 3. Mock infection of pregnant C57BL/6J control mice. (a) Percent weight gain of mid-gestation dams inoculated with Opt-MEM. (b) vRNA levels in maternal liver and spleen at harvest, measured by RT-qPCR at mid gestation. Each point represents an individual dam (n = 4 dams). (c) vRNA levels in matched placentas (circles) and fetuses (squares) from each dam, quantified by RT-qPCR at mid gestation. Each point represents an individual placenta or fetus derived from independent pregnancies. The dashed lines represent the limit of detection, ND = not detected.

Supplemental Figure 4. Placental infection frequency comparison between ancestral rOROV strain BeAn19991 and contemporary OROV isolate 2400023. Pooled vRNA levels of

placentas collected from early gestational infection (E4.5-7.5) compared between dams infected with strain BeAn19991 (n = 27 fetuses) and OROV 2400023 (n = 31 fetuses). Frequency of early gestation placental infection was significantly higher in the dams infected with OROV 2400023 (88.57% vs. 57.45%, χ^2 test, $p = 0.0022$). Overall placental vRNA levels tended to be higher in the 2400023-group than the BeAn19991-group (Mann–Whitney test, $p = 0.0650$).

Supplemental Figure 5. (a) Schematic of cross-mating between six-week-old IFNAR$^{-/-}$ females and WT C57BL/6J males to generate heterozygous pregnancies. (Created in BioRender. Tilston-lunel, N. (2026) https://BioRender.com/ ldb9voq). Pregnant dams were infected SC with rOROV BeAn19991 (n = 4 dams) or Opti-MEM (n = 3 dams) at E7.5 (early gestation). (b) vRNA levels in maternal liver and spleen at harvest (E14.5), measured by RT-qPCR. (c) vRNA levels in matched placentas (circles) and fetuses (squares) from each dam, quantified by RT-qPCR at early gestation. Each point represents an individual placenta or fetus derived from independent pregnancies. The dashed line represents the limit of detection, ND = not detected. (d) Infectious virus titrations of select placental (left) and fetal (right) homogenized tissues from a representative dam (D2), mean ± SD. The dashed line represents the limit of detection. (e) Immunofluorescence staining of select placental (left) and fetal (right) sections from dam D2, stained with anti-OROV antibody (red) and DAPI (blue). (f) Pooled fetal weights between mock-infected and rOROV BeAn19991-infected litters (Welch's t-test, p = 0.0653).

Supplemental Figure 6. Optimization of AM0059/88 M segment minigenome assay. BSRT7/5 cells were co-transfected with either increasing amounts of pTM1-N and constant amounts of pTM1-RdRp (250 ng) or increasing amounts of pTM1-RdRp and constant amounts of pTM1-N (250 ng), along with an AM0059/88 M-segment minigenome encoding humanized Renilla luciferase (hRenilla). A firefly luciferase-expressing plasmid, pTM1-FFLuc was also included in the transfection mixture to normalize for differences in transfection efficiency. Data are shown as relative luciferase activity (a) log10 scale and (b) linear scale. Each point represents an independent transfection (n = 3 independent biological replicates per condition), and data are presented as mean ± SE.

Supplemental Figure 7. High-throughput VNT assay set-up. (a) High-throughput VNT performed in a 96-well format using the same sera as in Fig. 6e. (b) LI-COR fluorescence scans (top) and crystal violet-stained plates (bottom) show a dose-dependent viral inhibition in samples from pups born to infected dams. Representative data shown from 3 dpi.

Supplemental Table: HCR probes used in this study.

Pair #	Initiator	Spacer	Probe	Probe	Spacer	Initiator
1	GTCCCTGCCTCTATATCT	tt	TACATCTACATTTGATCCGGAGGCA	CATTTTCAACGATGTACCACAACGG	tt	CCACTCAACTTTAACCCG
2	GTCCCTGCCTCTATATCT	tt	CAAGTGCTCAATGCTGGTGTTGTTA	TATGTGGCATTTGAAGCTAGATACG	tt	CCACTCAACTTTAACCCG
3	GTCCCTGCCTCTATATCT	tt	AAGATGTCTTACGTAAGACATCGAG	TCTTCTTCCTCAACCAAAAGAAGGC	tt	CCACTCAACTTTAACCCG
4	GTCCCTGCCTCTATATCT	tt	CCAATTTGCAATGGTTAATAACCAT	CATGGTTGACCTTACTTTTGGTGGG	tt	CCACTCAACTTTAACCCG
5	GTCCCTGCCTCTATATCT	tt	GACAACGGTCTTACCCTGCACCGTC	CCACAGTTCCAGTCGAATCCAGTGC	tt	CCACTCAACTTTAACCCG
6	GTCCCTGCCTCTATATCT	tt	CCCAGATGCGATCACCAATTAAGCA	CAGGATACCTAGCGCGCTGGGCCTT	tt	CCACTCAACTTTAACCCG
7	GTCCCTGCCTCTATATCT	tt	TTTGGCTGAGGTAAAGGGCTGTACT	TGAGTTCAGAGCCACTGTAGTAGTG	tt	CCACTCAACTTTAACCCG
8	GTCCCTGCCTCTATATCT	tt	TTTGCTGCTGGTGCTGAGATGTTCC	AATGATGGTGACGCAATGTACCTGG	tt	CCACTCAACTTTAACCCG
9	GTCCCTGCCTCTATATCT	tt	TTGAGATGCATAGGGTTCTCAAGGA	AAACATTCACTTTCTTCCCTTTGGT	tt	CCACTCAACTTTAACCCG
10	GTCCCTGCCTCTATATCT	tt	CCTCCGGCAACGGTATGGCCAAAAG	CATGGATGTCAACTTTATGAAGAAA	tt	CCACTCAACTTTAACCCG
11	GTCCCTGCCTCTATATCT	tt	GTTGCTGTAAGAGCAGCTTTTGAAG	GCCGAGCAATGGATGCGTGAAGAAA	tt	CCACTCAACTTTAACCCG
12	GTCCCTGCCTCTATATCT	tt	GATTCTCCCCAGCAGCAAGAGACTT	TAGGCACTCTGGCCTGGGCCAGAAC	tt	CCACTCAACTTTAACCCG

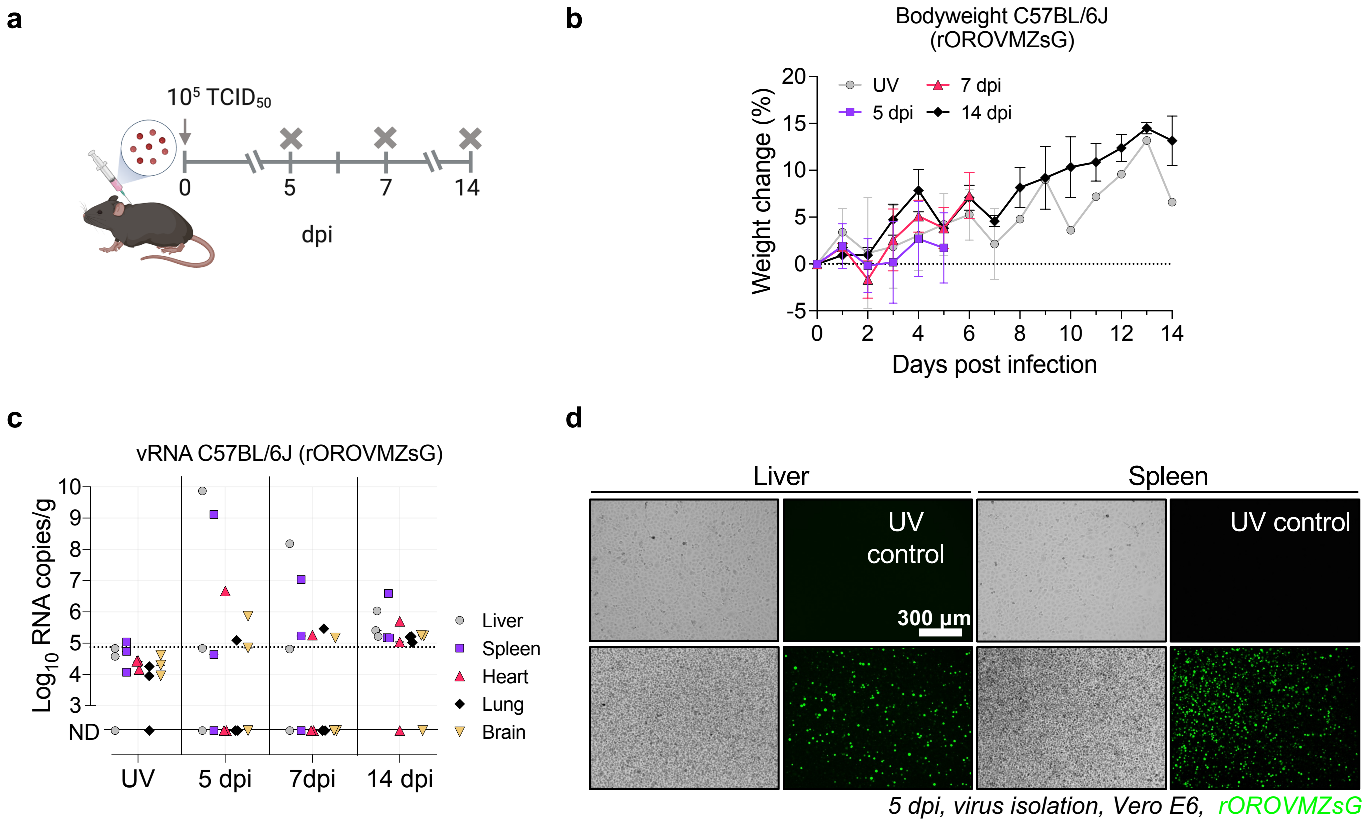

Supplementary Figure 1

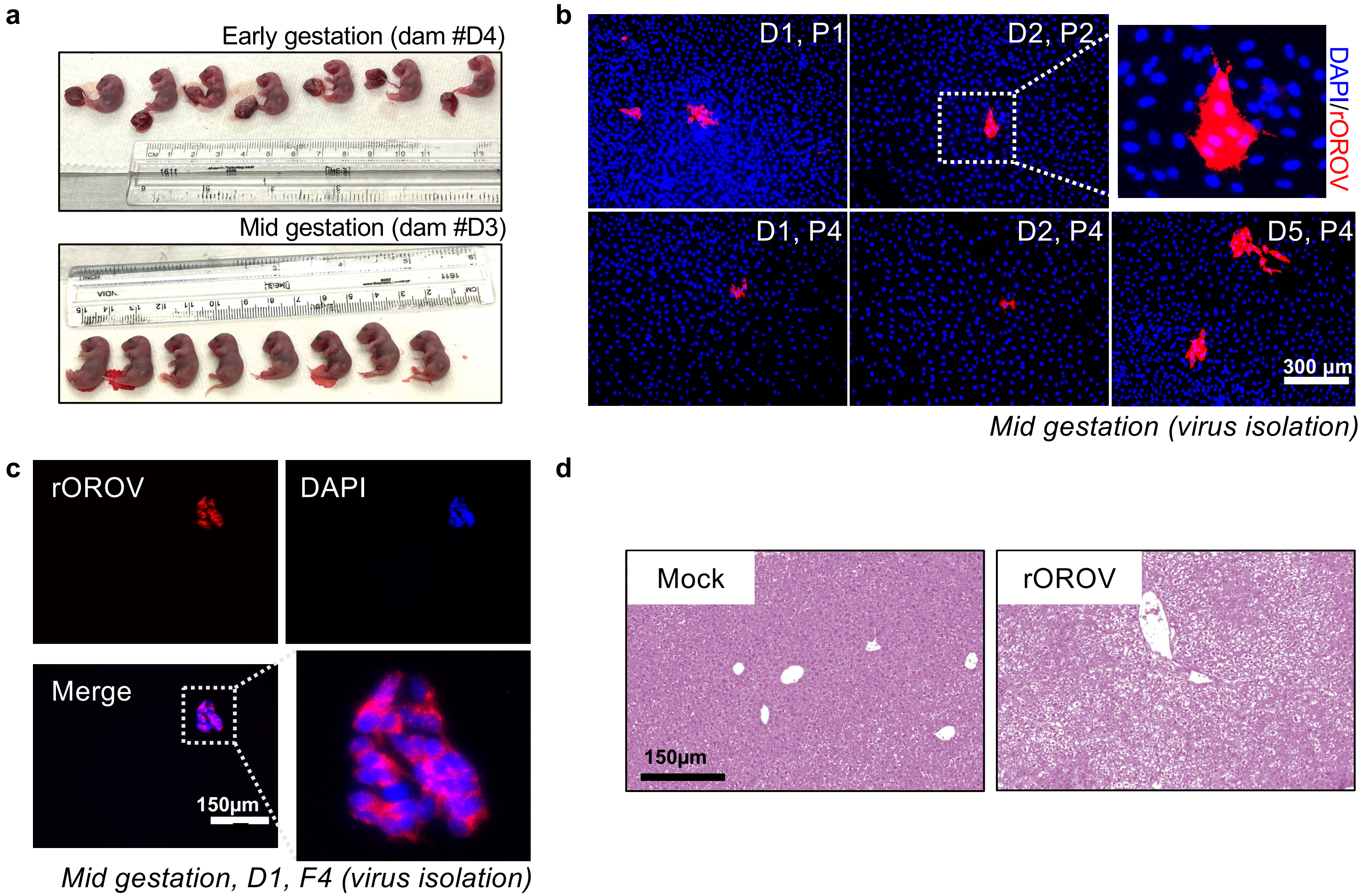

Supplementary Figure 2

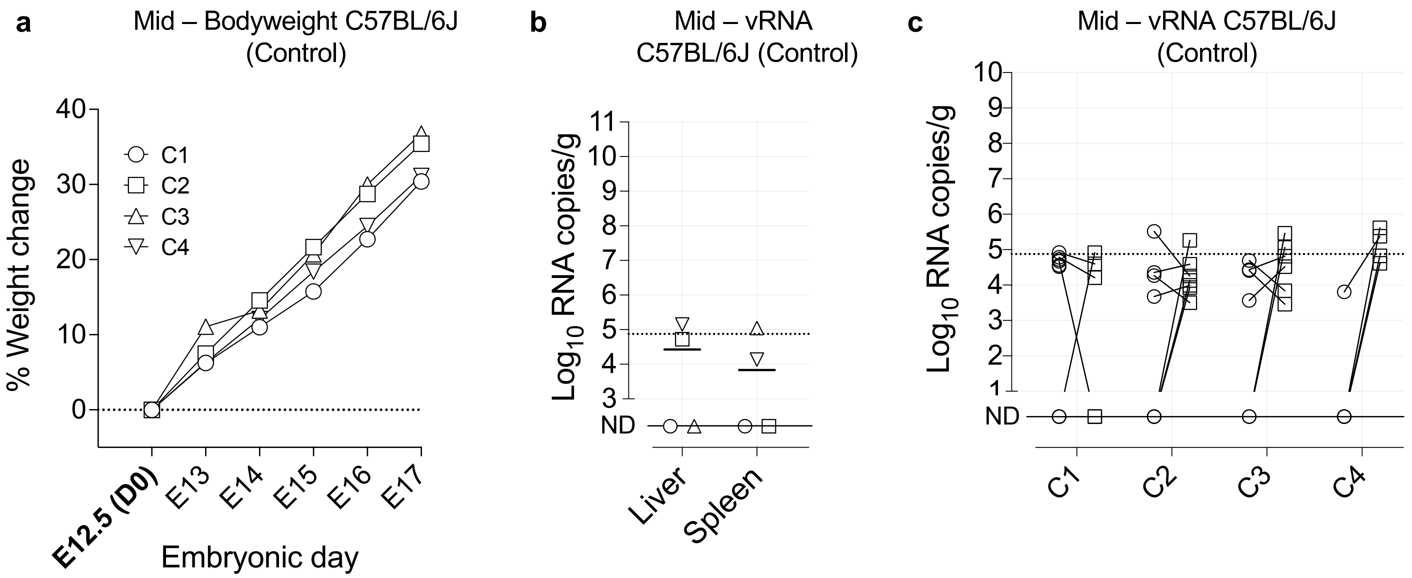

Supplementary Figure 3

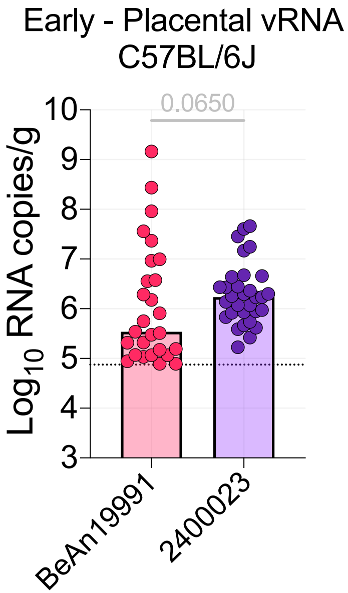

Supplementary Figure 4

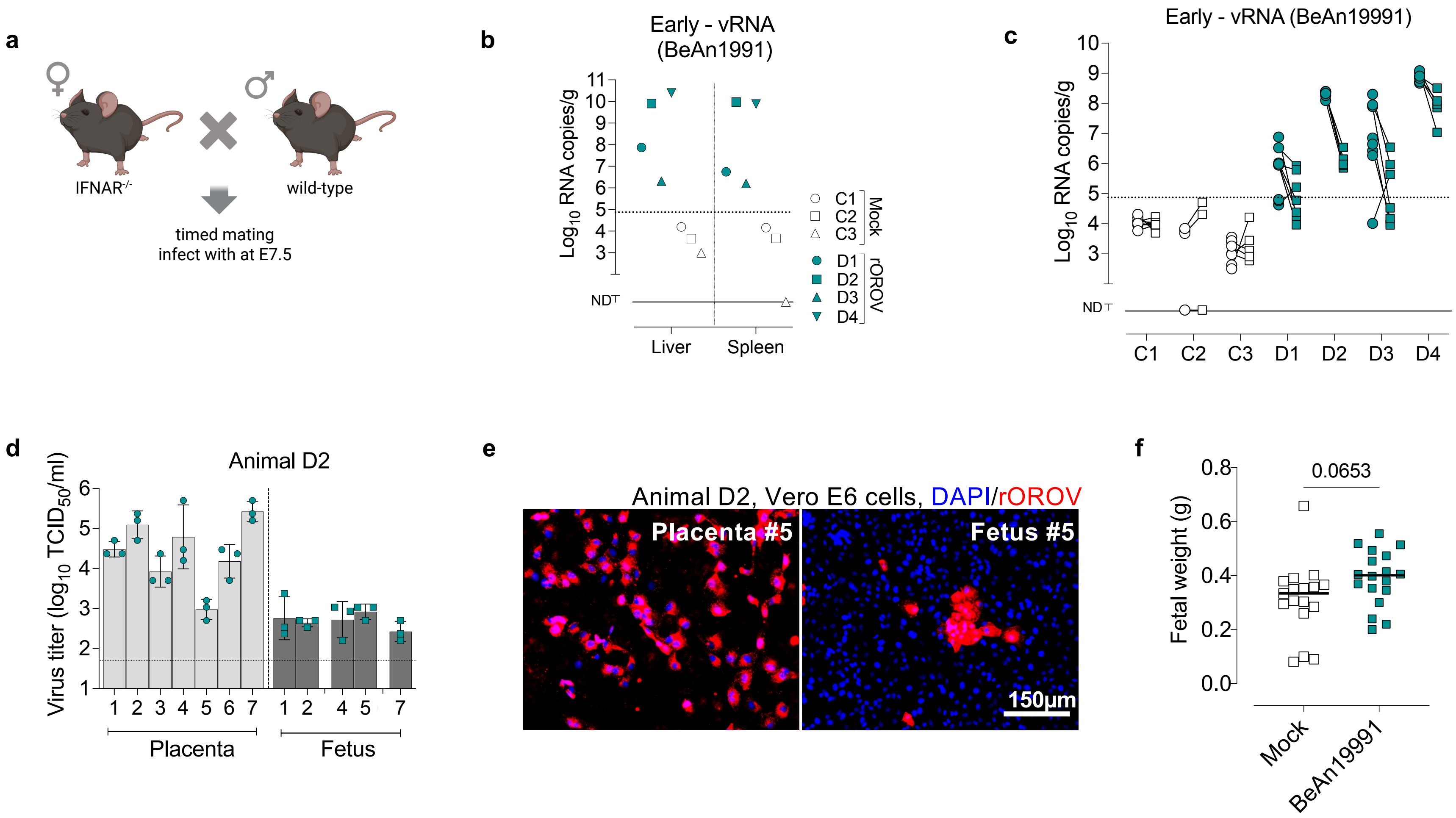

Supplementary Figure 5

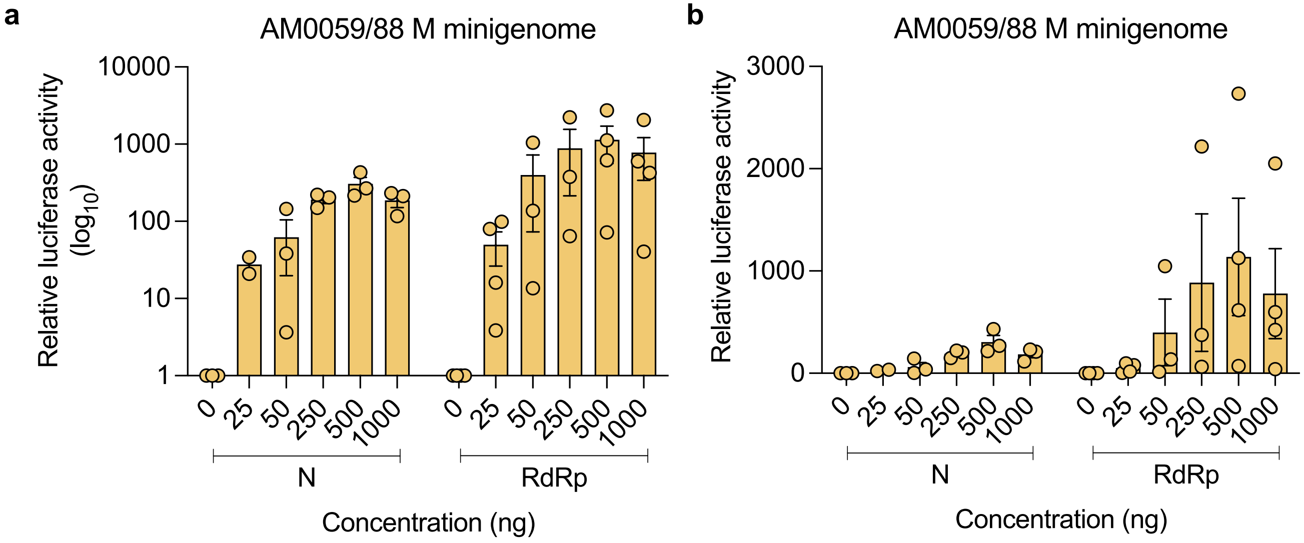

Supplementary Figure 6

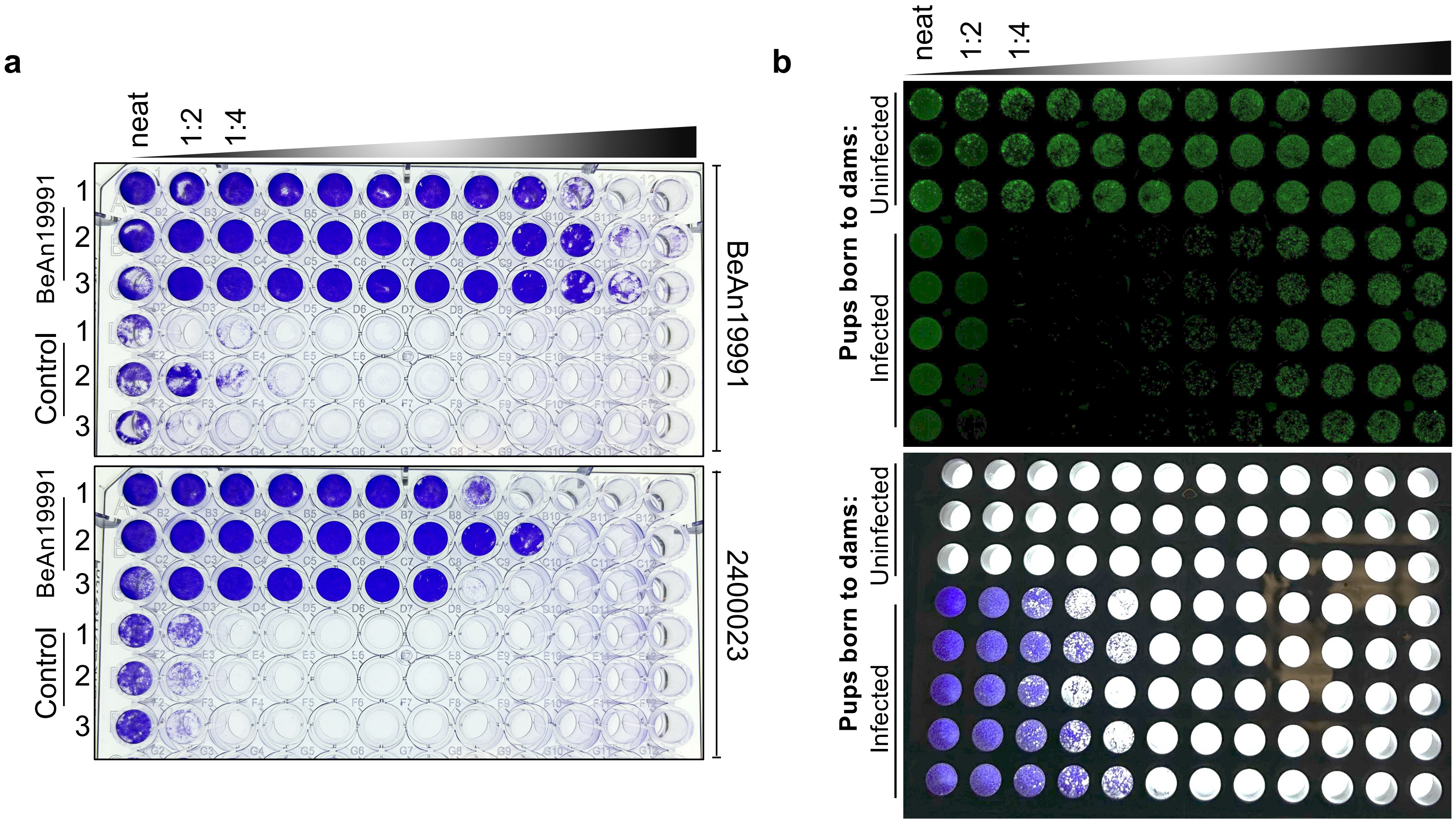

Supplementary Figure 7
