# Supplementary material for: Conserved pathogenesis of ancestral and contemporary Oropouche virus strains in a murine pregnancy model
Source: Nat Commun. 2026 May 12;17:6542. doi: 10.1038/s41467-026-72711-2 (PMC13381898; doi:10.1038/s41467-026-72711-2)
Supplement: Supplementary file 3 — Reporting Summary [file 41467_2026_72711_MOESM3_ESM.pdf]

nature portfolio

Corresponding author(s): Natasha Louise Tilston

Last updated by author(s): 2026/05/14

Reporting Summary

Nature Portfolio wishes to improve the reproducibility of the work that we publish. This form provides structure for consistency and transparency in reporting. For further information on Nature Portfolio policies, see our Editorial Policies and the Editorial Policy Checklist.

Statistics

For all statistical analyses, confirm that the following items are present in the figure legend, table legend, main text, or Methods section.

n/a	Confirmed	
☐	☒	The exact sample size (*n*) for each experimental group/condition, given as a discrete number and unit of measurement
☒	☐	A statement on whether measurements were taken from distinct samples or whether the same sample was measured repeatedly
☐	☒	The statistical test(s) used AND whether they are one- or two-sided<br>*Only common tests should be described solely by name; describe more complex techniques in the Methods section.*
☒	☐	A description of all covariates tested
☒	☐	A description of any assumptions or corrections, such as tests of normality and adjustment for multiple comparisons
☐	☒	A full description of the statistical parameters including central tendency (e.g. means) or other basic estimates (e.g. regression coefficient) AND variation (e.g. standard deviation) or associated estimates of uncertainty (e.g. confidence intervals)
☐	☒	For null hypothesis testing, the test statistic (e.g. *F*, *t*, *r*) with confidence intervals, effect sizes, degrees of freedom and *P* value noted<br>*Give P values as exact values whenever suitable.*
☒	☐	For Bayesian analysis, information on the choice of priors and Markov chain Monte Carlo settings
☒	☐	For hierarchical and complex designs, identification of the appropriate level for tests and full reporting of outcomes
☒	☐	Estimates of effect sizes (e.g. Cohen's *d*, Pearson's *r*), indicating how they were calculated

Our web collection on statistics for biologists contains articles on many of the points above.

Software and code

Policy information about availability of computer code

Data collection	Data were collected using standard laboratory methods, including RT–qPCR, virus titration assays, and fluorescence microscopy. No custom software was used for data collection.
Data analysis	GraphPad Prism (v10), Microsoft Excel and ImageJ were used for statistical analysis and data visualization.

For manuscripts utilizing custom algorithms or software that are central to the research but not yet described in published literature, software must be made available to editors and reviewers. We strongly encourage code deposition in a community repository (e.g. GitHub). See the Nature Portfolio guidelines for submitting code & software for further information.

Data

Policy information about availability of data

All manuscripts must include a data availability statement. This statement should provide the following information, where applicable:

- Accession codes, unique identifiers, or web links for publicly available datasets
- A description of any restrictions on data availability
- For clinical datasets or third party data, please ensure that the statement adheres to our policy

This study used publicly available sequences from GenBank, including contemporary OROV isolates from Brazil with the accession numbers: PP992525 (AM0088; S segment), PP992526 (AM0059; S segment), PP992527 (AM0088; M segment), PP992528 (AM0059; M segment), PP992529 (AM0088; L segment), and PP992530 (AM0059; L segment) as well as the prototype OROV strain BeAn19991: KP052852 (S segment), KP052851 (M segment) and KP052850 (L segment). Source data are provided with this paper. Additional information is available from the corresponding author (N.L.T.) upon reasonable request.

Research involving human participants, their data, or biological material

Policy information about studies with human participants or human data. See also policy information about sex, gender (identity/presentation), and sexual orientation and race, ethnicity and racism.

Reporting on sex and gender	n/a
Reporting on race, ethnicity, or other socially relevant groupings	n/a
Population characteristics	n/a
Recruitment	n/a
Ethics oversight	n/a

Note that full information on the approval of the study protocol must also be provided in the manuscript.

Field-specific reporting

Please select the one below that is the best fit for your research. If you are not sure, read the appropriate sections before making your selection.

☒ Life sciences ☐ Behavioural & social sciences ☐ Ecological, evolutionary & environmental sciences

For a reference copy of the document with all sections, see nature.com/documents/nr-reporting-summary-flat.pdf

Life sciences study design

All studies must disclose on these points even when the disclosure is negative.

Sample size	Sample sizes were determined using the resource equation method for animal experiments, which is appropriate when the expected effect size and variance are not known a priori and therefore formal power calculations are not feasible. For survival and in vivo outcome studies, we used a minimum of n = 5 mice per group, which yields an E value within the recommended range:<br>E = total number of animals − total number of groups.<br>For example, with 3 groups (control + two virus groups) and 5 animals/group, E = (3 × 5) − 3 = 12, which lies within the recommended 10 – 20 range for adequate error degrees of freedom. This group size is therefore expected to provide sufficient statistical interpretability while minimizing animal use.
Data exclusions	Females that did not establish pregnancy were excluded as maternal-fetal outcomes cannot be assessed in non-pregnant animals.<br>Following reviewer feedback during revision, animals that underwent viral rechallenge in the pathogenesis experiment were excluded from the final analysis to avoid confounding interpretation of primary infection outcomes. The revised analyses therefore include only primary infection cohorts.<br>No other data were excluded.
Replication	All experiments were performed using the same virus stock for each strain throughout the study. Virus stocks were generated, sequence-verified, and aliquoted prior to experimentation to ensure genetic stability and experimental consistency across cohorts. This approach ensured direct comparability of viral load, pathogenesis, and pregnancy outcomes across independent experiments.<br>Independent animal cohorts were used for each experiment, and observed infection and disease phenotypes were consistent across cohorts.<br>Technical assays were performed with appropriate technical replicates and independently repeated where indicated. All reported findings were successfully replicated.
Randomization	Animals were assigned to experimental groups at the time of infection to ensure balanced distribution across cohorts and litters where applicable. All animals meeting predefined inclusion criteria were included in the study.
Blinding	Investigators were not routinely blinded to group allocation during infection and outcome assessment. However, primary outcome measures, including viral load quantification by RT-qPCR and neutralization assays, were based on objective quantitative readouts using predefined analysis parameters. Where applicable, RT-qPCR analyses were performed without reference to clinical outcome. No subjective endpoints were used to determine inclusion in statistical analyses.

Reporting for specific materials, systems and methods

We require information from authors about some types of materials, experimental systems and methods used in many studies. Here, indicate whether each material, system or method listed is relevant to your study. If you are not sure if a list item applies to your research, read the appropriate section before selecting a response.

Materials & experimental systems

n/a	Involved in the study
☐	☒ Antibodies
☐	☒ Eukaryotic cell lines
☒	☐ Palaeontology and archaeology
☐	☒ Animals and other organisms
☒	☐ Clinical data
☒	☐ Dual use research of concern
☒	☐ Plants

Methods

n/a	Involved in the study
☒	☐ ChIP-seq
☒	☐ Flow cytometry
☒	☐ MRI-based neuroimaging

Antibodies

Antibodies used	anti-OROV (Oropouche virus immune ascitic fluid [V-505-701-562] (ATCC VR-1228AF), dilution 1:500<br>Alexa Fluor 594 goat anti-mouse IgG (H&L) (Invitrogen A11032, dilution 1:1000<br>Alexa Fluor 488 phalloidin (Invitrogen A12379), dilution 1:1000<br>4′,6-diamidino-2phenylindole (DAPI) nuclear stain (Fisher EN2248) , dilution 1:1000
Validation	The OROV immune ascitic fluid (ATCC VR-1228AF) has been previously characterized for detection of Oropouche virus antigen and has been used extensively in our laboratory and in my PhD work since 2011 for immunofluorescence-based detection of OROV. Specificity is supported by manufacturer documentation, prior published work, and consistent detection of viral antigen in infected samples with absence of signal in mock-infected controls processed in parallel.<br>Alexa Fluor-conjugated secondary antibodies (Invitrogen A11032 and A12379) are commercially validated by the manufacturer for species-specific detection of mouse IgG (H&L) in immunofluorescence applications and were used according to manufacturer recommendations.<br>DAPI (Fisher EN2248) was used as a nuclear counterstain following standard protocols.

Eukaryotic cell lines

Policy information about cell lines and Sex and Gender in Research

Cell line source(s)	Vero E6 (African green monkey kidney; ATCC CRL-1586) and A549 (human alveolar adenocarcinoma epithelial cells; ATCC CCL-185) were obtained directly from the American Type Culture Collection (ATCC).<br>BeWo (ATCC CCL-98), JEG-3 (ATCC HTB-36), and HTR-8 (HTR-8/SVneo; ATCC CRL-3271) cells were obtained from a collaborator; these lines originated from ATCC stocks.<br>BSR-T7/5 cells (baby hamster kidney–derived cells stably expressing T7 RNA polymerase) were obtained from a previously established laboratory source during postdoctoral training.
Authentication	Vero E6 and A549 cells were obtained directly from ATCC. BeWo, JEG-3, and HTR-8/SVneo cells were obtained from a collaborator and originated from ATCC stocks. BSR-T7/5 cells were obtained from a previously established laboratory source. Cell lines were not independently re-authenticated in this study.
Mycoplasma contamination	All cell lines were routinely tested for mycoplasma contamination using PCR-based detection assays and were confirmed negative prior to use in experiments.
Commonly misidentified lines<br>(See ICLAC register)	BeWo and JEG-3 are listed in the ICLAC register of commonly misidentified cell lines. These lines were used because they are well-established and widely accepted models of human trophoblast biology and placental infection.<br>No other cell lines used in this study are listed in the ICLAC register.

Animals and other research organisms

Policy information about studies involving animals; ARRIVE guidelines recommended for reporting animal research, and Sex and Gender in Research

Laboratory animals	C57BL/6J mice (6-week-old females; Jackson Laboratory, maintained in the IUSM colony) and IFNAR-/- mice (6-week-old males and females; Jackson Laboratory, maintained in the IUSM colony) were used. Mice were housed under specific pathogen-free conditions in a temperature- (20–24 °C) and humidity-controlled (40–60%) environment on a 12-hour light/dark cycle, with ad libitum access to food and water. C57BL/6J mice: Strain #000664, Jackson Laboratory; IUSM colony. IFNAR-/- mice: B6(Cg)-Ifnar1tm1.2Ees/J, strain #: 028288, Jackson Laboratory;IUSM colony.
Wild animals	This study did not involve wild animals.
Reporting on sex	Both male and female mice were used in this study, as indicated. For pregnancy experiments, timed matings were performed using female mice, and fetal outcomes were assessed at the litter level. Sex was not used as a biological variable in downstream analyses.
Field-collected samples	This study did not involve field-collected samples.

Ethics oversight	All animal work was performed in compliance with the Indiana University School of Medicine Institutional Animal Care and Use Committee (IACUC) under approved protocol #22080 (PI: Tilston). Experiments were conducted in an Animal Biosafety Level 2 (ABSL-2) facility in accordance with institutional and federal guidelines.

Note that full information on the approval of the study protocol must also be provided in the manuscript.

Plants

Seed stocks	n/a
Novel plant genotypes	n/a
Authentication	n/a
