# Supplementary material for: Conserved pathogenesis of ancestral and contemporary Oropouche virus strains in a murine pregnancy model
Source: Nat Commun. 2026 May 12;17:6542. doi: 10.1038/s41467-026-72711-2 (PMC13381898; doi:10.1038/s41467-026-72711-2)
Supplement: Supplementary file 2 — Peer Review file [file 41467_2026_72711_MOESM2_ESM.pdf]

nature portfolio

Peer Review File

Conserved pathogenesis of ancestral and contemporary Oropouche virus strains in a murine pregnancy model

Corresponding Author: Dr Natasha Tilston

This file contains all reviewer reports in order by version, followed by all author rebuttals in order by version.

Version 0:

Reviewer comments:

Reviewer #1

(Remarks to the Author)
Overall assessment:
The manuscript on Oropouche virus (OROV) infection in pregnancy employs a very interesting mouse model, which provides the possibility to test the general affinity of OROV to tissues in a pregnant mammal model. However, yet it lacks the necessary context and data to substantiate all its conclusions.
The major omissions concern the relevance of the model for the human situation (different pregnancy duration, different placentation etc), comparisons with related orthobunyaviruses (Schmallenberg virus, Akabane virus, Shuni virus), clear and transparent virological quantification, tissue tropism (especially in the central nervous system (CNS)) and also ustification of the used biosafety level.

Major issues:
Introduction/Context:
- The relevance and limitations of the murine model should be clarified (including pregnancy stages, timing, placentation' and vector context).
- Place OROV within the context of related viruses, such as Akabane virus and Schmallenberg virus, where congenital transmission, foetal malformations, brain infection and persistence are well documented.
- Explain the constellations of genome segments and the type of reassortment, emphasising the M segment and its implications for entry and neutralisation.
- Outline the expected outcomes of fetal infection and persistence based on these comparators.

Results/Data completeness:
- Report quantitative viral loads/titres (ranges, means and detection limits) for all tissues and foetuses; descriptions in text only are insufficient.
- Address the apparent lack of brain infection, despite the known CNS tropism of related viruses, and include differences in fetal vs. placental replication efficiency and IFN competence.
- Correlate histopathology with viral burden and include in situ hybridisation and broader staining panels.
- Sequence the virus in fetal tissues to detect adaptations to the fetal environment (especially in the S segment like NSs and also Gc. Is there frame shifts and/or deletions?).
- Ensure direct comparability with related viruses and prior experiments. This is completely missing.

Strains, passage history and statistics:

- Specify the passage history of ancestral and contemporary strains (cell culture versus suckling mouse/brain). Exclude artefacts of cell culture adaptation by comparing consensus sequences to the original stocks, and assess NSs/innate immune antagonism and other adaptive changes. This is a very critical point.
- Acknowledge the limitations of the IFNAR model and the small number of animals used, and include appropriate statistical analyses.
- Distinguish between passive maternal antibody transfer and active fetal immunization, and provide longitudinal pup data (including exact viral loads and brain persistence/clearance, as well as follow-up beyond maternal antibody waning). This is a major point missing in the study.
-

Discussion:
- Integrate evidence from ruminant diseases caused by related orthobunyaviruses (e.g. SBV in pregnant cattle and sheep) to provide a framework for transplacental infection, central nervous system (CNS) disease and malformations.
- Explore immune evasion and neutralisation by mapping Gc neutralising domains with monoclonal antibodies and including targeted in vitro assays.

Further points and biosafety issues:
- State explicitly at line 352 that the findings are from mouse models and are not yet generalizable.
- Line 375: Justify the use of BSL-2 for animal work and describe the midge-proofing measures employed. Note that BSL-3 is typically required in Europe for this vector-borne viruses. Please verify this and explain the safeguards in place.

Required additions:
- Tables/figures showing quantitative viral loads (maternal, placental, foetal and neonatal; include LOD/LOQ).
- ISH and expanded histopathology, and pathology–virology correlations.
- Deep sequencing of foetal/placental viruses and analysis of potential adaptive mutations (including NSs).
- Clear documentation of strain passage history and sequence identity to source stocks.
- A comparative discussion and, where feasible, in vitro neutralisation mapping with Gc-specific mAbs.
- Longitudinal foetal/pup follow-up to distinguish persistence versus clearance and passive versus active immunity.

Reviewer #2

(Remarks to the Author)
The manuscript “From prototype to outbreak: conserved pathogenesis of Oropouche virus in a novel murine pregnancy model highlights its public health implications” by Gunter et al. describes a series of experiments to compare an ancestral strain (BeAn19991) to contemporary strains (primarily 240023 and a minigenome experiment with AM0059/88) for their ability to infect immunocompetent adult mice, pregnant mice, cross the placental barrier, immunocompromised mice, and neutralization phenotypes. The authors conclude that vertical transmission is a shared feature of OROV strains, the modern OROV strains are more virulent than the ancestral strain, immune escape mutations have arisen in modern strains, and ancestral OROV infection in pregnancy can lead to maternal antibody protection in infected mouse pups. This area of research is highly significant given the ongoing OROV outbreak in South America, and pregnancy complications associated with OROV infections. Developing mouse models to study the mechanisms of vertical transmission is highly valuable to the field. While the studies do address some of these components, and found interesting results related to infection in maternal tissues and maternal antibody protection, the data supporting vertical transmission of both OROV strains in the immunocompetent model, higher pathogenicity and immune escape of the modern strain is weak and the conclusions are often overstated.

Major concerns:

1. The claim that both BeAn19991 and 240023 are readily vertically transmissible is not supported by the presented data. For the BeAn strain, only two fetuses from a single dam in the early infection and three birthed pups from a single dam in the mid infection have vRNA levels above detection, and for the 240023 strain only two fetuses from a single dam (maybe a third from a second dam, but it’s right on the line) have vRNA levels above detection (Figure 1 d,g,l). No evidence of infectious virus in fetuses is shown, and the one supporting IHC image of supposed lymphocytic infiltrate in a fetal brain does not have an uninfected control for comparison, and no information is given on what fetus this was taken from. The maternal infection claims are well supported, as infection is readily detectable in most livers and spleens for both OROV strains, and frequently and highly detected in placentas, however I do not believe the authors can make the statement that OROV efficiently crosses the maternal-fetal interface. In order to make these claims, the authors need to show infectious virus in fetal tissue.

2. In experiments relating to Figure 2, the authors perform replication kinetics assays in three different trophoblast cells and conclude that both the BeAn19991 strain and the 240023 strains replicate similarly in the placental cells and conclude that “these findings confirm that OROV is intrinsically capable of infecting placental cells and crossing the maternal–fetal barrier”. There are major concerns with the replication kinetics shown in Figure 2a-c. First, without having an early time point or inoculum titer, it is impossible to tell if the cells were actually infected with similar amounts of both viruses, and this data needs to be included for accurate assessment of replication kinetics. Additionally, based on the description in the figure legend and methods, it appears only a single replicate was performed. The legend does mention each point represents an independent replicate but there only appears to be one point per time – in which case it appears SD was calculated on technical replicates? This is not explained and therefore it appears only a single experiment was performed for each, and therefore at least two more independent replication kinetics assays, including a 1 hour or inoculum titer, need to be done. Additionally, while the data does clearly support that both OROV strains replicate well in the human trophoblast cells, no claims of crossing the placental barrier can be made based on this data.

3. In experiments related to Figure 4 and the authors’ claims that 240023 is more virulent/pathogenic than BeAn19991, with the lack of information on the number of animals used in the study and lack of statistics, it’s impossible to determine whether the data supports the claim that the 240023 strain is indeed more pathogenic/virulent than the ancestral strain in the IFNAR-/- model. No statistics are done on the survival curve in 4a, the point of the rechallenge is not clear and seems to artificially increase 240023’s virulence and decrease BeAn’s virulence since only one of the two survivors of BeAn at 14 dpi was re-challenged. Additionally the claims about high vRNA levels in tissues in 240023 “likely contributing to the more rapid disease progression observed in OROV 240023-infected mice” is not well supported because high vRNA loads were detected for both BeAn 19991 and 240023, except for the single BeAn survivor, and no statistics comparing vRNA loads

between BeAn19991 and 240023 seem to have been done.

4. The immune escape statement relating to Figure 5 is not well supported by the data. It is expected that anti-sera will better neutralize homologous virus vs. a heterologous virus. So the observation that BeAn antisera better neutralizes BeAn than 240023 is completely expected. To argue one-way immune escape, the opposite comparison of evaluating 240023 serum against BeAn19991 and 240023 needs to be done. If this was immune evasion adaptations, you would expect to see the 240023 serum neutralize the BeAn19991 similarly or better than 240023. If the 240023 serum neutralizes itself better than the BeAn19991 strain, it just suggests better homologous recognition of antigens than heterologous/cross-reactive recognition of antigens. This would be the expected outcome, because viruses tend to elicit stronger antibodies to homologous vs heterologous virus strains. Additionally, it is unclear where the serum came from - were these the IFNAR-/- mouse sera or C57BL/6 mouse sera in 5A? From non-pregnant mice or pregnant mice? 5B refers to maternal antibodies, but was the same sera used for A? This needs to be clarified.

Minor concerns:

• Supp Fig 1. needs images from the UV-inactivated controls to compare background fluorescence. D says representative shown, but from which mouse livers and spleen?

• Statement line 102: “By 14 dpi, vRNA loads had declined, consistent with viral clearance.” While days 5 and 7 have a select single sample from each tissue with high levels of detection, 1-2 of the samples for most tissues are below the limit of detection, whereas at 14 dpi, while the viral RNA amount is lower compared to the one really high sample at each day, there is a higher proportion of vRNA samples above the limit of detection (3/3 for liver and spleen vs only 1/3 or 2/3 for liver and spleen at 5 and 7 dpi). Is this really indicative of clearance, or slow viral replication? Hard to say with such small sample sizes. In support of saying this is a self-limiting infection, an additional later time point should be taken as well.

• Fig 1. The legend describes mock-inoculated pregnant dams as well, these should be included in b and e as a comparison for weight gain.

• Fig 1h and i) show the uninfected control comparison. In i, the figures are described as representative, but the dam and fetus these images were taken from should be stated.

• For fig 1 viral rna comparisons between early and late – include statistical analysis to support the claim.

• Negative control staining should be included for Figure 2. It’s also unclear why phalloidin was included, and only for the BeWo cells and not the others and this should be clarified.

• For figure 3 replication kinetics, why were A549 lung cells selected vs an IFN competent kidney epithelial cell line or a more biologically relevant cell type?

• Explain the difference between Figure 3 b and c.

• Text at line 210 says there are 3 differences between BeAn19991 and the contemporary strains in the 3’ UTR and references Figure 5e, should be 3e.

• Lines 241-243. The statements about AM0088 are confusing because the authors did not use AM0088 in their neutralization assays. It appears to be referencing a prior publication, but what those findings are and how it relates to the currents studies is not clear, especially regarding plaque morphology as there appears to be pretty minimal plaque morphology differences show in Figure 5d.

• No description of where the plasmids for the minigenome came from or how they were made. How are the two 0059/88 isolates combined? Lack of experimental details makes it difficult to assess what the results actually mean.

• The number of animals used in each study should be included.

Reviewer #3

(Remarks to the Author)
Oropouche virus is an understudied Orthobunyavirus even though human outbreaks have been reported in several countries in the Amazon region for the past 60 years. In the past ~2 years, Oropouche virus outbreaks have increased in both frequency and scope. Notably, reports of suspected Oropouche-associated vertical transmission, miscarriage, and congenital abnormalities have been documented raising the question, is Oropouche virus a threat to pregnancies in areas where the virus is endemic. Here, Gunter et al, use an immunocompetent mouse pregnancy model to evaluate Oropouche virus’s capacity to harm the developing fetus. Their data show that both ancestral and contemporary strains of Oropouche virus can infect the mouse placenta and be vertically transmitted, though all mice appeared to develop normally. These data suggest that Oropouche virus may have always had the capacity to cause congenital infections and that this is not an emergent property for the virus. This is perhaps not surprising because related viruses in the same family are well known for causing congenital infections, mostly in livestock. They also compared replicative fitness of the different strains in cell culture systems, neutralization potential, and virulence in immunocompromised mice. While there are some interesting findings

presented, enthusiasm is diminished by a lack of statistical analysis and overinterpretation of results. In addition, the presentation of the material is clear enough but not at the level of concision, precision, and crispness I would expect for a paper in this journal. As a result, there are a number of scientific issues and several things that need to be addressed to facilitate understanding by the reader. Specific comments follow.

Line 20: suggest adding context to “rise in congenital infections” to something like “rise in congenital infections associated with OROV cases.”

Line 29-30: I disagree that these data alone “conclusively confirm OROV as a vertically
30 transmissible arbovirus.” Rather they suggest, along with human epidemiological data, that OROV possesses worrisome properties that should prompt further investigation into the risk for pregnant people living in endemic areas.

Line 29-30: I would suggest here that emphasis should be on emphasizing that clinicians in areas endemic for OROV consider the possibility of congenital OROV infection, with countermeasure development etc. secondary.

Line 88-89: “congenital arboviral threat” is awkward, consider changing to something like teratogenic arbovirus or similar.

Line 99: why wasn’t serum assessed for vRNA loads?

Figure 1 and elsewhere: viral load plots indicate a limit of detection at 10^5 RNA copies/gram but there are points plotted below this line. Can the authors please explain this. Also, the limit of detection for the assay seems rather high, can the authors expand on why this is?

Figure 1 and in the text: I believe it would be easier for the reader to follow the text and the figure if the gestation timepoints were referred to as their corresponding embryonic day equivalents (E4.5, E7.5, or E12.5) rather than “early” or “mid” etc. Importantly, some justification should be provided as to why E4.5 and 7.5 data are pooled.

Figure 1: consider splitting this into 2 separate figures to facilitate larger panels.

Line 118: what timepoint is the necropsy timepoint?

Figure 1i: it would be helpful to include a panel from a similar region from the inactivated virus control group.

Line 148-151: These details are important but source information, etc is more appropriate for the methods. Here, I think it is more important to delineate how this specific strain compares genetically to the strains currently circulating in the Americas. Essentially, establishing a case that this strain is representative.

Line 157: onward, it is difficult to compare results with OROV 240023 to the recombinant BeAn19991because different inoculation timepoints were used: E4.7-7.5 and E12.5 for BeAn19991versus E7.5 and E14.5 for 240023. At a minimum this should be acknowledged as a limitation.

Figure S2: Why does infection of the dams appear to be so much less efficient for the E14.5 inoculations compared to everything else?

Line 142-146 and 162-164: It is important to acknowledge that the mouse placenta does not have a definitive structure until the midpoint of gestation: ~E10.5–11.5. With only limited evidence of fetal infection I don’t agree that there is sufficient evidence to support that OROV efficiently crosses the maternal-fetal barrier. This does not preclude the possibility of pathology developing as a result of placenta infection and the resultant placental insufficiency.

Line 163-164: it is unclear what exactly the authors mean by “with replication dynamics trending potentially better than the ancestral strain.” The authors do not present data on virus replication in Figure 1 and the associated supplementary figures referenced in this section, rather they include data on viral RNA loads in different tissues at different timepoints after inoculation.

Line 181: I don’t understand the rationale for testing replication kinetics and A549 cells vs. Vero E6 cells. While one may be IFN competent and one may be IFN deficient, this is still an apples to oranges comparison since A549 cells are a lung carcinoma cell line from a human and A549 cells are a kidney cell from an African Green Monkey.

Line 186: I don’t understand what the authors mean by “OROV AM0088 and AM0059 (treated together as AM0059/88.” Are these two separate viruses or a reassortant virus or something else completely? Or do they have identical M segment sequences so they are interchangeable in the mini genome assay described a few lines later. In sum, this section needs revision.

Line 187-189: move the lines about previously published studies to the discussion section.

Line 210: should the figure callout here be 3E not 5E?

Line 217-218: was statistical analysis done to compare the survival curves, vRNA loads in tissues, histopathology scores, etc? Without this it is not valid to state that the contemporary OROV isolate exhibits enhanced virulence. Further, the data in

C57BL/6 mice contradict this at least in the setting of pregnancy. Is the IFNAR1-/- model the most relevant model to assess these differences?

Figure 4A: images of neutralization plates are far less informative than showing a neutralization curve with NT50 or 90 values.

Line 233: what timepoint was immune sera harvested and from what mouse line?

Line 235 and Figure 5a and b: what statistical test was performed to determine that there was reduced neutralization of one strain versus the other? Further, what does “dilution factor of VNT” mean? Is this some endpoint titration at which the serum reduced virus levels by some percentage?

Line 235: I do not believe the authors present robust evidence to support the claim that there is antigenic divergence between these 2 OROV strains. Data are from n=3 animals.

Line 246-249: as noted elsewhere in this review, I do not agree with these statements.

Figure 5 would benefit from a survival curve. Additionally, what was the nAb titer in the animals at the timepoint in which they were challenged?

Line 281: the statement that the capacity for OROV for maternal-to-fetal transmission has been demonstrated needs a citation. It was this reviewer’s understanding that data were limited, with reports of suspected Oropouche-associated vertical transmission, miscarriage, and congenital abnormalities.

Line 293-294: This could be tested experimentally but without competition data there is not robust evidence to support this statement.

Line 307: neutralization data do not support the statement that there are antigenically distinct strains.

Line 426: MOI should have units associated with the value, e.g., PFU/cell or similar.

Discussion: overall the discussion would benefit from a paragraph about related orthobunyaviruses and congenital infection.

Version 1:

Reviewer comments:

Reviewer #1

(Remarks to the Author)
The manuscript has been revised in response to most of the points raised and has improved substantially.

However, several issues still need to be addressed:

- To better assess how suitable the mouse model is for studying the pathogenesis of this virus, the authors should discuss available data from related viruses, such as AKAV or SBV, for which both mouse and target-host data exist. This would help readers to better judge the value and limitations of mouse models and their transferability to the natural host species.

- Comparison with prior experiments, particularly OROV studies in mouse models (including the IFNAR-/- model), should be

provided. This point is still insufficiently addressed.

- Although additional sequencing data have now been included, whole-genome sequences e.g. from the fetal tissues are still missing. Providing these data remains recommended, as they could offer important insight into the role of fetal infection.

- The role of innate immunity is demonstrated in the mouse model. It should therefore be clearly stated that these findings cannot automatically be extrapolated to human infections (see lines 392-400).

- Lines 747-749: a supporting reference is missing for the statements.

- An additional ethics-related clarification is needed. In line 547, it is stated that all animals “succumbed to disease.” Please clarify that humane endpoints were applied and that animals were euthanized once these endpoints were reached.

Reviewer #2

(Remarks to the Author)
The revised manuscript has adequately addressed the concerns raised, including adding additional data as needed, tempering conclusions that were overstated, clarifying aspects of experiments that were originally unclear, and added appropriate controls and statistical analyses.

The results are of interest to the field and the results and conclusions are now supported by the data.

Reviewer #3

(Remarks to the Author)
The authors addressed most of my previous concerns. However, a few still remain:

Line 72-73: some references should be included here to support these statements.

Line 88: the references cited here do not directly address changes in Culicoides range, etc.

Line 90: “appears to be evolving” is contradictory to the findings presented here. The authors could say something like the clinical profile associated with Oropouche virus infection has expanded with the recent outbreaks to include pregnancy complications, neurological manifestations, etc. However, there are many reasons why this might be occurring, only one of which is that the virus has evolved new properties, so I would be careful how you frame this.

Line 139: I defer to the editor here but I would remove “novel” from the title and the text. There is nothing inherently novel with the mouse model since it is C57BL/6. The novelty stems from assessing the pathogenic potential of OROV in the setting of pregnancy not necessarily in the model used.

Line 306-309: can you include the differences between virus strains in a table in the supplementary files?

Line 93: the statement about IFN-I as a key barrier is not accurate. Since you mated WT sires with IFNAR1-/- dams the placentas and fetuses will have one intact Ifnar1 allele and thus maintain IFN signalling. It might not be as robust as with 2 WT animals mated but more experiments would be required to tease this apart.

Line 436: replace “live viruses” with something like “these virus isolates were not available from public repositories” or something similar.

Fig 6. Can the authors please clarify? Neutralization was assessed using immune sera collected at 5dpi (pregnant) and 14dpi (nonpregnant) and conclude that there is poor seroconversion against the homologous virus. However, these timepoints are rather early (particularly the 5dpi) to see evidence of robust seroconversion. Typically, one would need to wait 21-28 days.

Line 701: is this timeframe normal for mice for maternal transfer of Ab? Can you include a citation?. In humans and monkeys you have a window of about 3-6 months.

Response to Reviewer comment about RT-qPCR limit of detection: The authors responded with the following: “The apparent discrepancy arises because points plotted below the indicated LoD represent samples with detectable signal that fell below the quantitative range of our standard curve; these are shown for transparency but should be interpreted as below the quantitative threshold.” Based on this explanation is this not the “limit of quantification” which is different from the LoD. If you look at the MIQE guidelines (PMID: 19246619), the limit of detection is defined “as the lowest concentration at which 95% of the positive samples are detected. In other words, within a group of replicates containing the target at concentrations at the LoD, no more than 5% failed reactions should occur.” The lower limit of quantification is harder to define. As a heuristic I think it is okay to say that LLoQ is the same as the LoD, but I’m not sure it is “transparent” to show these because they should not be considered positive.

Open Access This Peer Review File is licensed under a Creative Commons Attribution 4.0 International License, which permits use, sharing, adaptation, distribution and reproduction in any medium or format, as long as you give appropriate credit to the original author(s) and the source, provide a link to the Creative Commons license, and indicate if changes were made.
In cases where reviewers are anonymous, credit should be given to 'Anonymous Referee' and the source.
The images or other third party material in this Peer Review File are included in the article's Creative Commons license, unless indicated otherwise in a credit line to the material. If material is not included in the article's Creative Commons license and your intended use is not permitted by statutory regulation or exceeds the permitted use, you will need to obtain permission directly from the copyright holder.
To view a copy of this license, visit https://creativecommons.org/licenses/by/4.0/

REVIEWER COMMENTS

Reviewer #1 (Remarks to the Author):

Overall assessment:

The manuscript on Oropouche virus (OROV) infection in pregnancy employs a very interesting mouse model, which provides the possibility to test the general affinity of OROV to tissues in a pregnant mammal model. However, yet it lacks the necessary context and data to substantiate all its conclusions.

The major omissions concern the relevance of the model for the human situation (different pregnancy duration, different placentation etc), comparisons with related orthobunyaviruses (Schmallenberg virus, Akabane virus, Shuni virus), clear and transparent virological quantification, tissue tropism (especially in the central nervous system (CNS)) and also justification of the used biosafety level.

We thank the reviewer for the constructive and detailed comments, which have helped us substantially improve the clarity, rigor, and context of our study. Below we provide a point-by-point response, with changes incorporated into the revised manuscript as indicated.

Major issues:

Introduction/Context:
- The relevance and limitations of the murine model should be clarified (including pregnancy stages, timing, placentation, and vector context).
We thank the reviewer for this suggestion. We have revised the limitations section of the Discussion to clarify the relevance of the murine pregnancy model, including differences in gestational timing, placentation, and maternal-fetal immune dynamics compared with human pregnancy. We also note that infections were performed using controlled timing and direct inoculation in the absence of natural vector-mediated transmission, which may influence viral kinetics and tissue tropism. These additions frame the murine model as a tractable but imperfect system for studying OROV vertical transmission and emphasize cautious extrapolation to human pregnancy. (Revised text: lines 1130 – 1140).

- Place OROV within the context of related viruses, such as Akabane virus and Schmallenberg virus, where congenital transmission, foetal malformations, brain infection and persistence are well documented.
We have added a new paragraph early in the Discussion, placing OROV within the context of related orthobunyaviruses such as Schmallenberg and Akabane viruses, where congenital infection and fetal CNS disease are well established, and explicitly contrast these outcomes with those observed in our murine OROV model. (Revised text: lines 1118 – 1129).

- Explain the constellations of genome segments and the type of reassortment, emphasising the M segment and its implications for entry and neutralisation.
We agree that clarification of genome segment constellations and reassortment is important. We have therefore added a statement to the Introduction noting that the current outbreak likely arose from complex reassortment among divergent OROV S, M, and L segment lineages, informed by large-scale genomic analyses. Detailed phylogenetic relationships and proposed reassortment events, including the observation that the M segment clusters with the most prevalent Brazilian lineage while the S and L segments group separately, are comprehensively described by Naveca

et al. (2024) and are cited accordingly to avoid overloading the Introduction with extensive phylogenetic detail. (Revised text: lines 163 – 171).

- Outline the expected outcomes of fetal infection and persistence based on these comparators.
We thank the reviewer for this suggestion. In related orthobunyaviruses such as Schmallenberg, Akabane, and Shuni viruses, congenital infection is frequently associated with fetal malformations, central nervous system involvement, and persistent infection, particularly in ruminant hosts. We have now added this context to the Discussion. (Revised text: lines 1118 – 1129).

<u>Results/Data completeness:</u>

- Report quantitative viral loads/titres (ranges, means and detection limits) for all tissues and foetuses; descriptions in text only are insufficient.
We agree and have revised the Results text throughout to explicitly report quantitative viral loads and titers, including ranges, means, and limits of detection, for all maternal tissues, placentas, and fetal samples. Numerical values are now stated directly in the text in addition to being shown in figures.

- Address the apparent lack of brain infection, despite the known CNS tropism of related viruses, and include differences in fetal vs. placental replication efficiency and IFN competence.
We thank the reviewer for raising this important point. We agree that several related bunyaviruses, and OROV in IFN-deficient settings, exhibit marked CNS tropism. We have now clarified this issue in the Discussion. Across both ancestral and contemporary strains, we consistently observed substantially higher vRNA loads in placental tissues compared to matched fetal tissues. We have now extensively discussed this in the results section (Revised section: lines 255 – 506). Importantly, our IFNAR$^{-/-}$ models demonstrate that loss of type I IFN signaling results in widespread fetal dissemination, including robust brain infection, confirming that the limited fetal CNS involvement observed in wild-type pregnancies reflects IFN-mediated restriction rather than an absence of intrinsic neurotropism. These points are now explicitly discussed in the revised manuscript (lines 1118 – 1207).

- Correlate histopathology with viral burden and include in situ hybridisation and broader staining panels.
We thank the reviewer for this thoughtful suggestion. Direct correlation of histopathology with viral burden within the same fetal and placental tissues was not feasible due to the study design, as whole placentas and two intact pups per litter were reserved for histological analysis, while the remaining tissues were processed separately for RT-qPCR.

Expanded in situ hybridization and broader immunohistochemical staining panels were not incorporated in the current study, which was designed to establish and characterize a murine model of congenital OROV infection rather than to define cell-type-specific localization of viral RNA. Importantly, the quantitative RT-qPCR data and histopathological findings provide complementary evidence supporting placental infection and fetal exposure. Detailed spatial mapping of viral RNA and cellular tropism will be pursued in future mechanistic studies building on this model.

- Sequence the virus in fetal tissues to detect adaptations to the fetal environment (especially in the S segment like NSs and also Gc. Is there frame shifts and/or deletions?).
We thank the reviewer for this insightful suggestion. Investigation of potential viral adaptation in fetal tissues, particularly in the NSs and glycoproteins, is important. The primary objective of this study, however, was to establish conserved vertical transmission in a murine model, rather than

to characterize intra-host viral evolution. To address the reviewer's concern regarding potential sequence changes, we aligned the NSs coding sequences of the parental inoculum and the recovered virus from the placental sample shown in Figure 1h and did not identify frameshifts, deletions, or obvious truncations (see alignment below). While deep sequencing of fetal-derived virus would be required to rigorously assess low-frequency variants or minor quasispecies, the viral loads detected in fetal tissues and the limited infection window constrain sensitivity for robust variant detection in the current study.

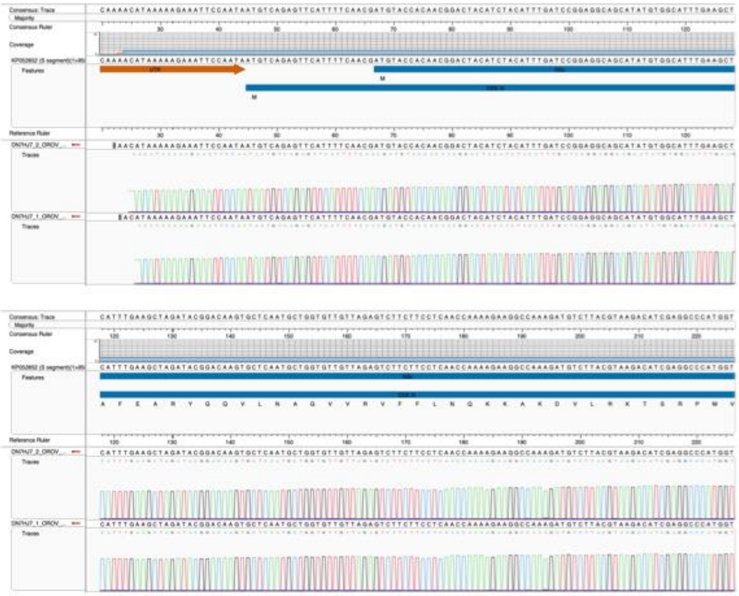

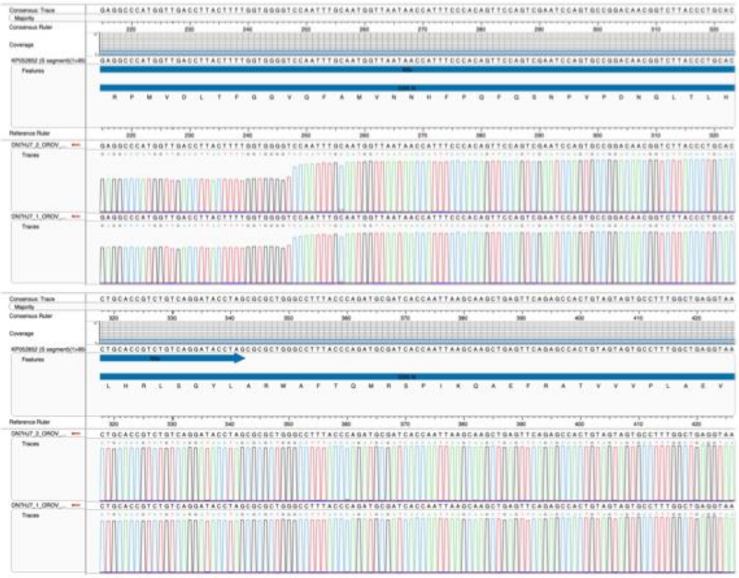

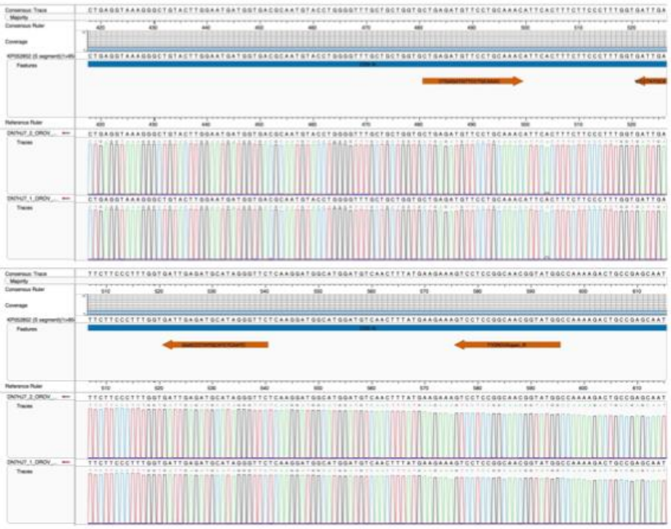

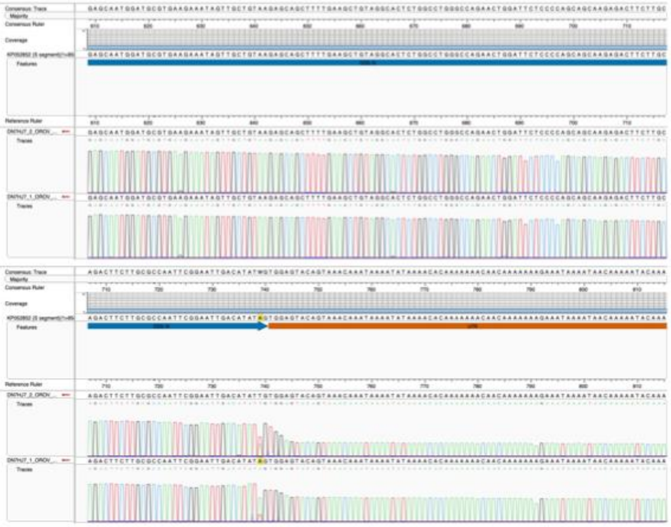

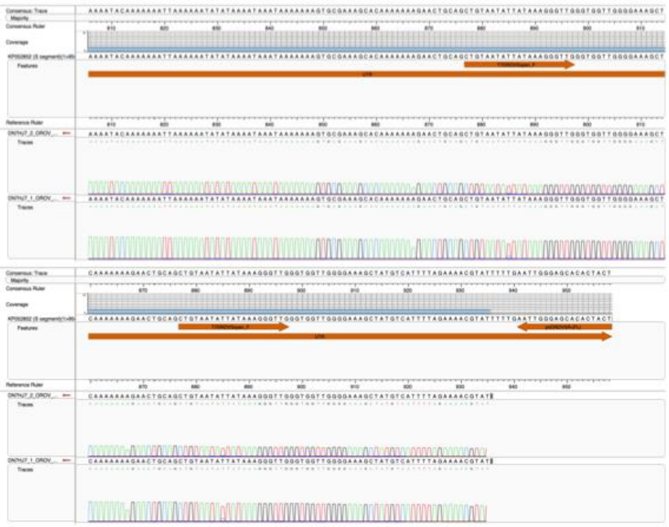

- Ensure direct comparability with related viruses and prior experiments. This is completely missing.

We appreciate the reviewer's comment and the opportunity to address it. Although we did not perform direct side-by-side experimental comparisons with ruminant-associated orthobunyaviruses (e.g., SBV or AKAV), our study design incorporates experimental frameworks and outcome measures that are directly aligned with prior congenital arbovirus models. These include timed gestational infection, quantitative assessment of placental and fetal viral burden, and analysis of maternal antibody transfer. This standardized experimental structure enables meaningful conceptual and mechanistic comparisons within the broader congenital orthobunyavirus literature.

Strains, passage history and statistics:

We thank the reviewer for this comment. We note that strain identity, source, and passage history are specified throughout the Methods, and all comparative experiments were performed using low-passage, sequence-verified virus stocks. Statistical analyses are reported with exact tests, p-values, and sample sizes, and individual data points are shown in all figures. For clarity, we have consolidated this information in the Methods section to explicitly state passage history and statistical approaches used across experiments.

- Specify the passage history of ancestral and contemporary strains (cell culture versus suckling mouse/brain). Exclude artefacts of cell culture adaptation by comparing consensus sequences to the original stocks, and assess NSs/innate immune antagonism and other adaptive changes. This is a very critical point.

We thank the reviewer for raising this important point. The passage history and provenance of all viral strains are described in the Methods; however, we have revised this section to further clarify these details. rOROV BeAn19991 and rOROVMZsG were generated by reverse genetics and propagated twice in Vero E6 cells. The contemporary isolate OROV 240023 was obtained from the UTMB Arbovirus Reference Collection, passaged three times in Vero E6 cells prior to receipt, and once upon arrival in our laboratory, followed by plaque purification to generate clonal stocks. No viruses used in this study were passaged in suckling mice or brain tissue. All working stocks were low-passage, sequence-verified, and titrated in parallel. No unexpected mutations were identified in any of our stocks.

With respect to NSs-mediated innate immune antagonism, sequencing confirmed intact NSs coding regions in all viral stocks. Moreover, viral sequences recovered from the placenta show in Figure 1h did not reveal NSs alterations, arguing against selection of adaptive variants during infection. Consistent with preserved NSs function, the ancestral strain exhibited the expected phenotype in IFNAR$^{-/-}$ mice. While detailed mechanistic dissection of NSs antagonism is an important area for future investigation, the present study was designed to evaluate vertical transmission dynamics and comparative pathogenesis during pregnancy.

- Acknowledge the limitations of the IFNAR model and the small number of animals used, and include appropriate statistical analyses.

We appreciate this point and have added a statement acknowledging that the IFNAR$^{-/-}$ model, while highly permissive for OROV, does not reflect intact IFN signaling. We also recognize that group sizes were modest. Animal numbers were determined a priori in accordance with institutional ethical guidelines and the resource equation method (Charan & Kantharia, 2013), which is appropriate when effect sizes are unknown. For example, in survival experiments involving three groups (control plus two virus groups) with five animals per group (total n = 15), the calculated E value is 12 (E = total animals – total groups = 15 - 3), which falls within the recommended range of 10 - 20.Where appropriate, statistical analyses have now been included and are described in the Methods. We have clarified this in the revised manuscript.

- Distinguish between passive maternal antibody transfer and active fetal immunization, and provide longitudinal pup data (including exact viral loads and brain persistence/clearance, as well as follow-up beyond maternal antibody waning). This is a major point missing in the study.
We thank the reviewer. We believe this point is already addressed in Figure 7. Our longitudinal follow-up clearly demonstrates that pups born to OROV-exposed dams exhibit early, short-lived protection associated with high neutralizing titers that wane over time, consistent with passive maternal antibody transfer. In contrast, pups from naïve dams developed neutralizing responses only after infection, indicating de novo antibody induction. Together, the viral load data (Fig. 7b-d) and neutralization kinetics (Fig. 7e-g) distinguish passive maternal protection from active pup immunity.

Discussion:

- Integrate evidence from ruminant diseases caused by related orthobunyaviruses (e.g. SBV in pregnant cattle and sheep) to provide a framework for transplacental infection, central nervous system (CNS) disease and malformations.
As suggested, we have now expanded our Discussion to compare our findings with congenital infection and malformations described for Schmallenberg, Akabane, and Shuni viruses in ruminants. This comparative framework highlights both shared features (placental infection) and key differences (the absence of CNS malformations in our model).

- Explore immune evasion and neutralisation by mapping Gc neutralising domains with monoclonal antibodies and including targeted in vitro assays.
We agree that immune evasion and antibody-mediated neutralisation are central aspects of OROV biology. In this study, we addressed these questions by evaluating cross-neutralisation between ancestral and contemporary strains using polyclonal sera and by assessing maternal antibody transfer and protection in pups (Figures 6 and 7). These experiments provide functional insight into immune protection and antigenic divergence in a biologically relevant in vivo setting.

Detailed mapping of Gc neutralising epitopes using monoclonal antibodies and targeted *in vitro* assays would further refine our understanding of antigenic determinants. However, the present study was designed to examine vertical transmission and comparative pathogenesis during pregnancy, with neutralisation analyses incorporated to contextualize observed strain differences. We have revised the Discussion to more explicitly situate our findings within the framework of established Gc-directed antibody responses in related orthobunyaviruses and to link Gc sequence divergence with the reduced neutralisation observed for the contemporary isolate. (Revised text: lines 951 – 1038).

Further points and biosafety issues:

- State explicitly at line 352 that the findings are from mouse models and are not yet generalizable.
We have revised this text to clearly state that our findings are specific to the murine model and cannot yet be generalized to humans.

- Line 375: Justify the use of BSL-2 for animal work and describe the midge-proofing measures employed. Note that BSL-3 is typically required in Europe for this vector-borne viruses. Please verify this and explain the safeguards in place.
We acknowledge that OROV is classified as a BSL-3 pathogen in Europe. However, in the United States OROV is designated as a Biosafety Level 2 (BSL-2) agent according to the *Biosafety in Microbiological and Biomedical Laboratories, 6th Edition (BMBL6)* and CDC guidance. All in vivo work described in this manuscript was therefore performed at BSL-2 in strict compliance with Indiana University School of Medicine's Institutional Biosafety Committee (IBC) and Institutional

Animal Care and Use Committee (IACUC) protocols. To minimize the risk of accidental arthropod exposure, all animal housing and procedure rooms are fully contained within an ABSL-2 facility that is insect-secure, with double-door access, sealed windows, air curtains, and restricted entry. No vectors are present in the facility, and all cages are microisolator type to further prevent arthropod escape. These safeguards ensure that work with OROV was conducted safely and in compliance with U.S. regulations.

Required additions:

- Tables/figures showing quantitative viral loads (maternal, placental, foetal and neonatal; include LOD/LOQ).
Where appropriate, we now include representative quantitative ranges in the Results text to further aid interpretation.

- ISH and expanded histopathology, and pathology–virology correlations.
In situ hybridization, expanded histopathology, and direct pathology-virology correlations were not included, as these analyses were not feasible within the constraints of the current study design, which was focused on establishing and characterizing a murine model of congenital OROV infection.

- Deep sequencing of foetal/placental viruses and analysis of potential adaptive mutations (including NSs).
Deep sequencing of fetal and placental virus populations was not undertaken, as the study was designed to evaluate vertical transmission potential rather than intra-host evolution. Viral loads and short infection windows further limit sensitivity for low-frequency variant detection. Analysis of fetal-derived viral diversity will be addressed in future studies.

- Clear documentation of strain passage history and sequence identity to source stocks.
This information has now been provided.

- A comparative discussion and, where feasible, in vitro neutralisation mapping with Gc-specific mAbs.
We agree that mapping neutralising epitopes using Gc-specific monoclonal antibodies would provide important mechanistic insight into antigenic determinants and strain-specific differences. The present study was designed to evaluate vertical transmission dynamics, pregnancy outcomes, and functional cross-neutralisation between ancestral and contemporary isolates. Accordingly, we focused on biologically relevant polyclonal neutralisation assays and in vivo protection. We have revised the Discussion to provide a comparative context.

- Longitudinal foetal/pup follow-up to distinguish persistence versus clearance and passive versus active immunity.
We thank the reviewer. Longitudinal pup follow-up data are already presented in Figure 6, which demonstrates transient protection consistent with passive maternal antibody transfer rather than active fetal immunization.

Reviewer #2 (Remarks to the Author):

The manuscript “From prototype to outbreak: conserved pathogenesis of Oropouche virus in a novel murine pregnancy model highlights its public health implications” by Gunter et al. describes a series of experiments to compare an ancestral strain (BeAn19991) to contemporary strains (primarily 240023 and a minigenome experiment with AM0059/88) for their ability to infect

immunocompetent adult mice, pregnant mice, cross the placental barrier, immunocompromised mice, and neutralization phenotypes. The authors conclude that vertical transmission is a shared feature of OROV strains, the modern OROV strains are more virulent than the ancestral strain, immune escape mutations have arisen in modern strains, and ancestral OROV infection in pregnancy can lead to maternal antibody protection in infected mouse pups. This area of research is highly significant given the ongoing OROV outbreak in South America, and pregnancy complications associated with OROV infections. Developing mouse models to study the mechanisms of vertical transmission is highly valuable to the field. While the studies do address some of these components, and found interesting results related to infection in maternal tissues and maternal antibody protection, the data supporting vertical transmission of both OROV strains in the immunocompetent model, higher pathogenicity and immune escape of the modern strain is weak and the conclusions are often overstated.

We thank the reviewer for their careful and thoughtful evaluation of our manuscript. We appreciate the recognition of the significance of developing models to study OROV vertical transmission and maternal antibody protection. We have carefully considered all of the reviewers' concerns and have revised the manuscript to clarify our claims, strengthen statistical analyses, provide additional methodological detail and controls, and temper interpretations where data do not fully support stronger conclusions. We believe these changes address the reviewer's comments and improve the clarity and rigor of the manuscript.

Major concerns:

1. The claim that both BeAn19991 and 240023 are readily vertically transmissible is not supported by the presented data. For the BeAn strain, only two fetuses from a single dam in the early infection and three birthed pups from a single dam in the mid infection have vRNA levels above detection, and for the 240023 strain only two fetuses from a single dam (maybe a third from a second dam, but it's right on the line) have vRNA levels above detection (Figure 1 d,g,l). No evidence of infectious virus in fetuses is shown, and the one supporting IHC image of supposed lymphocytic infiltrate in a fetal brain does not have an uninfected control for comparison, and no information is given on what fetus this was taken from. The maternal infection claims are well supported, as infection is readily detectable in most livers and spleens for both OROV strains, and frequently and highly detected in placentas, however I do not believe the authors can make the statement that OROV efficiently crosses the maternal-fetal interface. In order to make these claims, the authors need to show infectious virus in fetal tissue.
We thank the reviewer for this careful and thoughtful assessment and agree that fetal infection occurs at lower frequency and abundance than placental infection in immunocompetent C57BL/6J dams. We have revised the manuscript to clarify this distinction and to avoid overstating the efficiency of fetal infection. Our data demonstrate robust and reproducible maternal infection, with frequent and high-level vRNA detection in maternal liver and spleen tissues. vRNA levels in placentas following both early- and mid-gestational infection are higher than in the fetal tissues, which were sporadic and occurred at lower levels, and were observed in a limited number of fetuses per litter. We now explicitly state in the revised manuscript that fetal infection is infrequent under immunocompetent conditions. Importantly, we note that infectious virus was successfully isolated from fetal tissue in one instance, confirming that productive fetal infection can occur, albeit rarely, in immunocompetent pregnancies. This observation is consistent with a recent study of OROV vertical transmission, which similarly reported rare recovery of infectious virus and emphasized that successful virus isolation generally requires high viral burdens. We have revised the manuscript to explicitly acknowledge the low frequency of fetal virus isolation in immunocompetent dams and do not claim efficient fetal replication under these conditions

Crucially, additional evidence for the intrinsic capacity of OROV to cross the maternal-fetal interface is provided by our IFNAR$^{-/-}$ crossbred pregnancy model. In this setting, loss of maternal type I interferon signaling resulted in uniformly high placental viral loads and consistent detection of infectious virus in both placental and fetal tissues. These findings demonstrate that OROV is intrinsically capable of breaching the maternal-fetal barrier, while identifying host interferon signaling as a critical determinant restricting fetal dissemination in immunocompetent pregnancies. Finally, with respect to histopathology, we have elected to remove all histopathological analyses from the revised manuscript to avoid overinterpretation.

2. In experiments relating to Figure 2, the authors perform replication kinetics assays in three different trophoblast cells and conclude that both the BeAn19991 strain and the 240023 strains replicate similarly in the placental cells and conclude that "these findings confirm that OROV is major concerns with the replication kinetics shown in Figure 2a-c. First, without having an early time point or inoculum titer, it is impossible to tell if the cells were actually infected with similar amounts of both viruses, and this data needs to be included for accurate assessment of replication kinetics. Additionally, based on the description in the figure legend and methods, it appears only a single replicate was performed. The legend does mention each point represents an independent replicate but there only appears to be one point per time – in which case it appears SD was calculated on technical replicates? This is not explained and therefore it appears only a single experiment was performed for each, and therefore at least two more independent replication kinetics assays, including a 1 hour or inoculum titer, need to be done. Additionally, while the data does clearly support that both OROV strains replicate well in the human trophoblast cells, no claims of crossing the placental barrier can be made based on this data.
We thank the reviewer for raising these important points regarding the interpretation and presentation of the replication kinetics experiments shown in Figure 2, which is now Figure 3 in the revised manuscript. We agree that clarification of experimental design, replicates, and the conclusions drawn from these data is warranted and have revised the manuscript accordingly. First, regarding the concern about equivalent infection at early time points, both BeAn19991 and 240023 viruses were used to infect trophoblast cells at identical multiplicities of infection (MOI), calculated based on $TCID_{50}$ titers determined on Vero E6 cells. To address the reviewer's concern, we have now added our inoculum data. These data demonstrate that both viruses establish infection at similar initial levels in all three trophoblast cell types. Second, with respect to biological replicates, we acknowledge that the original legend was unclear. The replication kinetics were from three biological replicates, not technical replicates. We have revised the figure legend to avoid ambiguity regarding replicate number and variance calculation. We agree with the reviewer that these data alone do not demonstrate placental barrier crossing. Accordingly, we have revised the Results text to clarify that the trophoblast infection experiments are intended to assess cell-intrinsic permissiveness and replication competence of OROV strains in placental-derived cells, rather than trans-barrier transmission. Claims regarding maternal-fetal interface crossing are now restricted to *in vivo* pregnancy models, where placental and fetal tissues were directly examined.

3. In experiments related to Figure 4 and the authors' claims that 240023 is more virulent/pathogenic than BeAn19991, with the lack of information on the number of animals used in the study and lack of statistics, it's impossible to determine whether the data supports the claim that the 240023 strain is indeed more pathogenic/virulent than the ancestral strain in the IFNAR-/- model. No statistics are done on the survival curve in 4a, the point of the rechallenge is not clear and seems to artificially increase 240023's virulence and decrease BeAn's virulence since only one of the two survivors of BeAn at 14 dpi was re-challenged. Additionally, the claims about high vRNA levels in tissues in 240023 "likely contributing to the more rapid disease progression observed in OROV 240023-infected mice" is not well supported because high vRNA loads were

detected for both BeAn 19991 and 240023, except for the single BeAn survivor, and no statistics comparing vRNA loads between BeAn19991 and 240023 seem to have been done.
We thank the reviewer for this careful critique and agree that the original presentation required clarification regarding animal numbers, statistical analysis, and conclusions drawn from the IFNAR$^{-/-}$ infection experiments. We have revised both the figure and the Results text to address these concerns directly. First, we have updated Figure 4 (now Figure 5) to remove secondary challenge experiments entirely. All animals in both BeAn19991- and 240023-infected groups now succumbed to primary infection, eliminating any ambiguity introduced by differential rechallenge and ensuring direct comparability between strains. The revised figure clearly indicates animal numbers per group, which are now explicitly stated in both the figure legend and Methods. We have also revised the Results text to remove claims that OROV 240023 is more virulent than BeAn19991 in our IFNAR$^{-/-}$ model. Instead, we now state that both strains cause rapid, severe disease in IFNAR$^{-/-}$ mice.

4. The immune escape statement relating to Figure 5 is not well supported by the data. It is expected that anti-sera will better neutralize homologous virus vs. a heterologous virus. So the observation that BeAn antisera better neutralizes BeAn than 240023 is completely expected. To argue one-way immune escape, the opposite comparison of evaluating 240023 serum against BeAn19991 and 240023 needs to be done. If this was immune evasion adaptations, you would expect to see the 240023 serum neutralize the BeAn19991 similarly or better than 240023. If the 240023 serum neutralizes itself better than the BeAn19991 strain, it just suggests better homologous recognition of antigens than heterologous/cross-reactive recognition of antigens. This would be the expected outcome, because viruses tend to elicit stronger antibodies to homologous vs heterologous virus strains. Additionally, it is unclear where the serum came from - were these the IFNAR-/- mouse sera or C57BL/6 mouse sera in 5A? From non-pregnant mice or pregnant mice? 5B refers to maternal antibodies, but was the same sera used for A? This needs to be clarified.
We thank the reviewer for this careful and constructive critique and agree that homologous virus is expected to be neutralized more efficiently than heterologous virus. Our analysis focused on sera raised against the ancestral strain (rOROV BeAn19991), which shows reduced cross-neutralization of OROV 240023 relative to homologous virus. As the reviewer correctly notes, this comparison alone is insufficient to demonstrate directional immune escape, as stronger neutralization of homologous virus is the expected outcome in most viral systems. We have therefore revised the text to describe these findings more conservatively as reduced cross-neutralization sensitivity of 240023 relative to BeAn19991, rather than as evidence of one-way immune evasion. We do not interpret these data as demonstrating directional immune escape, but rather as indicating measurable antigenic differences consistent with sequence polymorphisms in structural proteins (Now revised Figure 6; revised text, lines 755 – 774).

We have also clarified the source of sera used in Figure 6 (Methods and figure legend). All neutralization assays were performed using sera from immunocompetent C57BL/6J mice. These included sera from non-pregnant mice collected at 14 dpi and pregnant mice infected at mid-gestation and collected at 5 dpi. No IFNAR$^{-/-}$ sera were used in these neutralization experiments.

Minor concerns:

• Supp Fig 1. needs images from the UV-inactivated controls to compare background fluorescence. D says representative shown, but from which mouse livers and spleen?
This has now been included, and information on panel D has also been included.

• Statement line 102: “By 14 dpi, vRNA loads had declined, consistent with viral clearance.” While

days 5 and 7 have a select single sample from each tissue with high levels of detection, 1-2 of the samples for most tissues are below the limit of detection, whereas at 14 dpi, while the viral RNA amount is lower compared to the one really high sample at each day, there is a higher proportion of vRNA samples above the limit of detection (3/3 for liver and spleen vs only 1/3 or 2/3 for liver and spleen at 5 and 7 dpi). Is this really indicative of clearance, or slow viral replication? Hard to say with such small sample sizes. In support of saying this is a self-limiting infection, an additional later time point should be taken as well.
We thank the reviewer for this comment and agree that, given the small sample sizes and inter-individual variability, it is not possible to definitively distinguish viral clearance from low-level persistence based solely on these time points. We have therefore revised the manuscript to avoid overinterpreting these data. Specifically, we have modified the text to remove the statement that declining vRNA levels at 14 dpi are *"consistent with viral clearance"* (Revised text, line 245). While we agree that additional later time points would further strengthen conclusions regarding infection resolution, we believe the revised text appropriately reflects the data as presented.

• Fig 1. The legend describes mock-inoculated pregnant dams as well, these should be included in b and e as a comparison for weight gain
We have now included mock-inoculated pregnant C57BL/6J dams as controls. These data are presented in Supplementary Figure 3, which shows maternal weight gain across gestation, as well as the absence of detectable viral RNA in maternal tissues from mock-inoculated animals. We chose to include these data in the Supplementary Information to maintain clarity in the main figures while ensuring that relevant controls are fully available for comparison. The main text and figure legends have been updated accordingly.

• Fig 1h and i) show the uninfected control comparison. In i, the figures are described as representative, but the dam and fetus these images were taken from should be stated.
These images have now been removed from the revised manuscript.

• For fig 1, viral RNA comparisons between early and late – include statistical analysis to support the claim.
We thank the reviewer for this suggestion. In the revised manuscript, we have restructured Figure 1 and split it into two figures. We have also modified the accompanying text. Figure 2 now been early, mid, and late gestation data. We no longer make statistical claims directly comparing early versus late maternal viral RNA levels in Figure 1. Instead, the data are presented to illustrate stage-specific infection patterns, with statistical analyses applied only where direct comparisons are biologically and experimentally appropriate. The revised text reflects this change and no longer requires early-versus-late statistical testing to support the stated conclusions.

• Negative control staining should be included for Figure 2. It's also unclear why phalloidin was included, and only for the BeWo cells and not the others and this should be clarified.
We thank the reviewer and have clarified this point. Phalloidin was included only for BeWo cells to demonstrate that the cells used in these experiments were not syncytialised. As BeWo cells can undergo spontaneous or forskolin-induced syncytialisation, phalloidin staining was used to visualize actin filaments and confirm that the cells remained mononuclear under our experimental conditions. This was not necessary for the other cell lines, which do not undergo syncytialisation. Negative control staining for all cell types has now been included.

• For figure 3 replication kinetics, why were A549 lung cells selected vs an IFN competent kidney epithelial cell line or a more biologically relevant cell type?

We selected A549 lung epithelial cells because they are a well-established, IFN-competent human cell line commonly used in orthobunyavirus studies, allowing direct comparison with prior work. We agree that other epithelial cell types could also be informative. Importantly, our aim here was to compare replication between OROV strains under identical IFN-competent conditions. Any differences in replication efficiency, particularly those driven by variation in NSs function, would have been apparent in this system, as previously published bunyavirus studies have demonstrated.

• Explain the difference between Figure 3 b and c.
Figure 3b and 3c (now Figure 4 b and 4c) address distinct but complementary questions. Figure 3b compares transcriptional output of BeAn19991 and AM0059/88 M-segment minigenomes when paired with their cognate polymerases, demonstrating intrinsic differences in activity. Figure 3c controls for polymerase identity by pairing both minigenomes with BeAn19991 RdRp, showing that the enhanced activity of the AM0059/88 M segment persists independently of polymerase sequence. We have clarified this distinction in the revised text.

• Text at line 210 says there are 3 differences between BeAn19991 and the contemporary strains in the 3' UTR and references Figure 5e, should be 3e.
This has now been corrected.

• Lines 241-243. The statements about AM0088 are confusing because the authors did not use AM0088 in their neutralization assays. It appears to be referencing a prior publication, but what those findings are and how it relates to the currents studies is not clear, especially regarding plaque morphology as there appears to be pretty minimal plaque morphology differences show in Figure 5d.
We thank the reviewer for highlighting this ambiguity and agree that clarification was needed. AM0088 was not directly tested in our neutralization or plaque assays due to the unavailability of infectious virus stocks. We have revised the text to explicitly state that functional experiments were performed exclusively using BeAn19991 and the contemporary Cuban isolate OROV 240023. References to AM0088 are intended solely to provide sequence-level and contextual support, based on prior published studies, for interpreting observed antigenic differences within the M segment. We do not intend to imply direct functional comparison between AM0088 and BeAn19991 in this study. We have also revised the text to avoid overstating plaque morphology differences, which were modest and not statistically significant under the conditions tested. The revised manuscript now clearly distinguishes between experimentally derived data (BeAn19991 vs 240023) and sequence-based contextual comparisons to AM0088.

• No description of where the plasmids for the minigenome came from or how they were made. How are the two 0059/88 isolates combined? Lack of experimental details makes it difficult to assess what the results actually mean.
The origin and construction of all minigenome plasmids, including how sequence information from the AM0059/88 isolates was incorporated, are now clearly described in the expanded Methods section (Plasmids and minigenome assay).

• The number of animals used in each study should be included.
This has now been included.

Reviewer #3 (Remarks to the Author):

Oropouche virus is an understudied Orthobunyavirus even though human outbreaks have been reported in several countries in the Amazon region for the past 60 years. In the past ~2 years,

Oropouche virus outbreaks have increased in both frequency and scope. Notably, reports of suspected Oropouche-associated vertical transmission, miscarriage, and congenital abnormalities have been documented raising the question, is Oropouche virus a threat to pregnancies in areas where the virus is endemic. Here, Gunter et al, use an immunocompetent mouse pregnancy model to evaluate Oropouche virus's capacity to harm the developing fetus. Their data show that both ancestral and contemporary strains of Oropouche virus can infect the mouse placenta and be vertically transmitted, though all mice appeared to develop normally. These data suggest that Oropouche virus may have always had the capacity to cause congenital infections and that this is not an emergent property for the virus. This is perhaps not surprising because related viruses in the same family are well known for causing congenital infections, mostly in livestock. They also compared replicative fitness of the different strains in cell culture systems, neutralization potential, and virulence in immunocompromised mice. While there are some interesting findings presented, enthusiasm is diminished by a lack of statistical analysis and overinterpretation of results. In addition, the presentation of the material is clear enough but not at the level of concision, precision, and crispness I would expect for a paper in this journal. As a result, there are a number of scientific issues and several things that need to be addressed to facilitate understanding by the reader. Specific comments follow.

We thank the reviewer for their thoughtful and detailed assessment of our manuscript. We appreciate the recognition of the importance of studying OROV vertical transmission and agree that improving precision, clarity, and statistical support will strengthen the work. In response to the reviewer's comments, we have revised the manuscript to: (i) temper and clarify statements regarding vertical transmission and antigenic divergence, (ii) provide additional statistical analyses for viral loads, survival, and neutralization assays, (iii) clarify methodological details (serum sources, assay design, MOI units, embryonic day timing and virus strain designations), (iv) improve figure presentation (LOD explanation, gestational timepoint labels, control images, neutralization curves with NT50 values), and (v) revise the language for concision and precision. We believe these changes substantially improve the rigor and readability of the manuscript and address the reviewer's concerns.

Line 20: suggest adding context to "rise in congenital infections" to something like "rise in congenital infections associated with OROV cases."
This line has now been revised.

Line 29-30: I disagree that these data alone "conclusively confirm OROV as a vertically 30 transmissible arbovirus." Rather, they suggest, along with human epidemiological data, that OROV possesses worrisome properties that should prompt further investigation into the risk for pregnant people living in endemic areas.
We thank the reviewer for this important point and agree that the original wording in the Abstract overstates the conclusions. The Abstract has now been revised to more accurately reflect the our findings.

Line 29-30: I would suggest here that emphasis should be on emphasizing that clinicians in areas endemic for OROV consider the possibility of congenital OROV infection, with countermeasure development etc. secondary.
As suggested for the Abstract, these concluding statements have been modified to place greater emphasis on the clinical relevance of our findings, particularly the need for heightened awareness of potential congenital OROV infection in endemic regions, while explicitly situating the experimental data alongside emerging epidemiological evidence.

Line 88-89: “congenital arboviral threat” is awkward, consider changing to something like teratogenic arbovirus or similar.
We thank the reviewer for this helpful suggestion. The phrase “congenital arboviral threat” has been removed and the sentence revised to more clearly and accurately reflect the study’s focus on OROV infection during pregnancy and its relevance to maternal and fetal health.

Line 99: Why wasn’t serum assessed for vRNA loads?
In this study, our focus was specifically on tissue compartments (maternal organs, placenta, and fetal tissues) to assess viral replication and tropism at the maternal-fetal interface. While vRNA would likely be detectable in serum during acute infection, our experimental design prioritized direct analysis of infected tissues rather than circulation. We agree that including serum vRNA loads could provide additional insight into systemic infection dynamics, and we highlight this as a useful future direction.

Figure 1 and elsewhere: viral load plots indicate a limit of detection at 10^5 RNA copies/gram but there are points plotted below this line. Can the authors please explain this. Also, the limit of detection for the assay seems rather high, can the authors expand on why this is?
We thank the reviewer for this comment. The apparent discrepancy arises because points plotted below the indicated LoD represent samples with detectable signal that fell below the quantitative range of our standard curve; these are shown for transparency but should be interpreted as below the quantitative threshold. The relatively high LoD (10^5 RNA copies/gram) reflects the normalization to tissue mass, where RNA extracted from small samples is scaled to copies per gram. The underlying RT-qPCR assay has a sensitivity comparable to other published viral load assays, but when expressed per gram of tissue, the LoD appears higher. We have clarified this in the Methods.

Figure 1 and in the text: I believe it would be easier for the reader to follow the text and the figure if the gestation timepoints were referred to as their corresponding embryonic day equivalents (E4.5, E7.5, or E12.5) rather than “early” or “mid” etc. Importantly, some justification should be provided as to why E4.5 and 7.5 data are pooled.
We thank the reviewer for this helpful suggestion. We agree that referring to gestational stages by embryonic day improves clarity. In the revised manuscript and figures, we now explicitly annotate all infection time points using their corresponding embryonic day equivalents (E4.5, E7.5, E12.5), while retaining the terms “early,” “mid,” and “late” gestation for readability and consistency across figures.

With respect to pooling E4.5 and E7.5 data, these time points were grouped because both represent pre-placentation stages of pregnancy, prior to full establishment of the mature hemochorial placenta in mice. At these stages, viral exposure occurs before placental barrier formation, and preliminary analyses revealed comparable maternal viral burdens and placental outcomes between E4.5 and E7.5 infections. Pooling these time points, therefore, increased statistical power without obscuring biologically meaningful differences.

Figure 1: consider splitting this into 2 separate figures to facilitate larger panels.
We thank the reviewer for this suggestion. Figure 1 has now been split into two separate figures to facilitate larger panels and improve clarity and readability.

Line 118: what timepoint is the necropsy timepoint?
We thank the reviewer for this clarification request. All dams were necropsied at embryonic day (E) 17.5. Dams infected during early gestation (E7.5) were therefore assessed at 9.5 days post-infection, whereas dams infected during mid gestation (E12.5) were assessed at 4.5 days post-

infection. This information has now been added to the Results section to clarify the timing of tissue collection.

Figure 1i: it would be helpful to include a panel from a similar region from the inactivated virus control group.
We thank the reviewer for this suggestion. To avoid overinterpretation and ensure consistency across conditions, we have removed this histopathology panel from the revised manuscript rather than expanding the figure with additional control images.

Line 148-151: These details are important but source information, etc is more appropriate for the methods. Here, I think it is more important to delineate how this specific strain compares genetically to the strains currently circulating in the Americas. Essentially, establishing a case that this strain is representative.
We thank the reviewer for this important suggestion. We agree that strain source information is more appropriate for the Methods section and have relocated these details accordingly. In the Results, we have revised the text to emphasize the genetic relevance of strain 240023, clarifying that it represents a contemporary outbreak-associated isolate circulating in Cuba that is closely related to the Brazilian OROV strains (AM0088, for instance), while harboring a limited number of coding differences. These differences are explicitly referenced and summarized in Fig. 6.

Line 157: onward, it is difficult to compare results with OROV 240023 to the recombinant BeAn19991 because different inoculation timepoints were used: E4.7-7.5 and E12.5 for BeAn19991 versus E7.5 and E14.5 for 240023. At a minimum this should be acknowledged as a limitation.
We thank the reviewer for highlighting this point. We agree that, in the original submission, the use of different gestational inoculation windows complicated direct comparison between rOROV BeAn19991 and OROV 240023. To address this concern, we have substantially revised the organization of the data and figures. Specifically, we have split the original Figure 1 into two separate figures (as suggested), presenting BeAn19991 and OROV 240023 infections independently. In addition, we have now performed and included a new mid-gestation infection experiment for OROV 240023 (E12.5), aligning this condition directly with the BeAn19991 mid-gestation model. These new data are presented in the revised Figure 2 and allow for a more appropriate comparison of maternal, placental, and fetal outcomes at matched embryonic stages. The revised text has been updated to reflect these changes.

Figure S2: Why does infection of the dams appear to be so much less efficient for the E14.5 inoculations compared to everything else?
We believe that the reduced maternal viral RNA levels observed following E14.5 inoculation may reflect limited time for systemic viral replication prior to sampling, rather than reduced infection efficiency. Late-gestation infections were necessarily assessed over a shorter window (approximately 3-4 days before euthanasia), which may be insufficient for OROV to reach peak maternal viral burdens, particularly in immunocompetent hosts (as we have shown in our non-pregnant mice).

Line 142-146 and 162-164: It is important to acknowledge that the mouse placenta does not have a definitive structure until the midpoint of gestation: ~E10.5–11.5. With only limited evidence of fetal infection I don't agree that there is sufficient evidence to support that OROV efficiently crosses the maternal-fetal barrier. This does not preclude the possibility of pathology developing as a result of placenta infection and the resultant placental insufficiency.
We thank the reviewer for highlighting the developmental timing of placental maturation in the mouse. We agree that the definitive labyrinthine placenta is not fully established until

approximately E10.5 - E11.5, and we have revised the manuscript to explicitly acknowledge this point. In the revised text, we avoid implying efficient fetal infection during early gestation and instead emphasize that viral RNA detection at these stages primarily reflects maternal and placental infection. Consistent with the reviewer's assessment, detection of viral RNA in fetal tissues under immunocompetent conditions was infrequent and occurred at low abundance. We have therefore modified our language throughout the Results and Discussion to clarify that our data do not demonstrate efficient fetal replication in immunocompetent dams. Rather, our findings support the conclusion that OROV readily infects the maternal compartment and placenta. Importantly, evidence that OROV is intrinsically capable of crossing the maternal-fetal interface is now provided by the IFNAR$^{-/-}$ crossbred pregnancy model, in which loss of maternal type I IFN signaling resulted in uniformly high placental viral loads and consistent detection of virus in fetal tissues.

Line 163-164: it is unclear what exactly the authors mean by "with replication dynamics trending potentially better than the ancestral strain." The authors do not present data on virus replication in Figure 1 and the associated supplementary figures referenced in this section, rather they include data on viral RNA loads in different tissues at different timepoints after inoculation.
We thank the reviewer for highlighting this lack of clarity. We agree that the phrase "with replication dynamics trending potentially better than the ancestral strain" was imprecise and not directly supported by the data presented. We have therefore removed this wording in the revised manuscript and now describe these data more accurately as viral RNA loads measured across tissues and timepoints, without inferring replication dynamics.

Line 181: I don't understand the rationale for testing replication kinetics and A549 cells vs. Vero E6 cells. While one may be IFN competent and one may be IFN deficient, this is still an apples to oranges comparison since A549 cells are a lung carcinoma cell line from a human and A549 cells are a kidney cell from an African Green Monkey.
We thank the reviewer for this comment. Our rationale for including both A549 (human IFN-competent lung carcinoma) and Vero E6 (African green monkey IFN-deficient kidney epithelial) cells was to provide a comparison of replication in the presence or absence of intact type I interferon responses. We agree that these lines differ in tissue origin, and we acknowledge this limitation in the revised text. Importantly, our goal was not to directly compare replication kinetics between the two cell types, but rather to evaluate whether strain-specific differences emerged under IFN-competent versus IFN-deficient conditions.

Line 186: I don't understand what the authors mean by "OROV AM0088 and AM0059 (treated together as AM0059/88." Are these two separate viruses or a reassortant virus or something else completely? Or do they have identical M segment sequences so they are interchangeable in the mini genome assay described a few lines later. In sum, this section needs revision.

We thank the reviewer for this comment and agree that the original phrasing was unclear. OROV AM0088 and AM0059 are distinct viral isolates; however, they possess identical M segment sequences and are therefore functionally interchangeable in assays assessing M segment-encoded functions. We have revised the manuscript to explicitly state this rationale.

Line 187-189: move the lines about previously published studies to the discussion section.
We thank the reviewer for this suggestion. The lines referring to previously published studies have been removed from the Results section.

Line 210: should the figure callout here be 3E not 5E?
This has now been corrected.

Line 217-218: was statistical analysis done to compare the survival curves, vRNA loads in tissues, histopathology scores, etc? Without this it is not valid to state that the contemporary OROV isolate exhibits enhanced virulence. Further, the data in C57BL/6 mice contradict this at least in the setting of pregnancy. Is the IFNAR1-/- model the most relevant model to assess these differences?
We thank the reviewer for this important comment. We agree that the original wording could be interpreted as implying enhanced virulence of the contemporary isolate, in a limited model setting. In the revised manuscript, we have removed statements suggesting increased virulence and have reframed this section to emphasize that overall disease kinetics, survival, and tissue viral RNA loads were comparable between BeAn19991 and OROV 240023 in the $IFNAR^{-/-}$ model, while also reporting any differences that we did observe. Survival curves were compared descriptively, as all animals in both groups uniformly succumbed to infection within a narrow time window. Tissue viral RNA loads were quantified and are now presented without inferential statements regarding replication dynamics or virulence. We further clarify that the $IFNAR^{-/-}$ model was used to assess intrinsic pathogenic potential under highly permissive conditions and not to infer differences in virulence relevant to immunocompetent or pregnant hosts. In the revised manuscript, we also discuss the differences observed between BeAn19991 and OROV 240023 in the C57BL/6 model following testing of their antibody responses.

Figure 4A: images of neutralization plates are far less informative than showing a neutralization curve with NT50 or 90 values.
We thank the reviewer for this suggestion and agree that representative plate images can be less informative than quantitative neutralization curves. In the revised manuscript, the plate images previously shown in Figure 4A have been moved to the Supplementary Information, and we now present neutralization curves derived from CPE-based virus neutralization assays. Semi-quantitative CPE scores were converted to percent neutralization values and fit using a variable-slope nonlinear regression model to visualize trends in neutralizing activity. Details for this are provided in the methods sections of the revised manuscript.

Line 233: what timepoint was immune sera harvested and from what mouse line?
We thank the reviewer for this question. Immune sera used for neutralization assays were collected from C57BL/6J mice. For gestational infections, sera were harvested at E17.5 (5 days post-infection following inoculation at E12.5), while sera from non-pregnant mice were collected at 14 days post-infection. These details have now been clarified in the revised manuscript.

Line 235 and Figure 5a and b: what statistical test was performed to determine that there was reduced neutralization of one strain versus the other? Further, what does “dilution factor of VNT” mean? Is this some endpoint titration at which the serum reduced virus levels by some percentage?
We thank the reviewer for this comment. The “dilution factor of VNT” refers to an endpoint virus neutralization titer, defined as the highest reciprocal serum dilution at which virus-induced cytopathic effect was reduced relative to virus-only controls. In these assays, serum was serially diluted and incubated with virus prior to infection, and neutralization was scored based on CPE. Endpoint dilution values, therefore, reflect the maximal dilution at which functional neutralizing activity is still detectable. This definition has now been clarified in the revised Methods and figure legends to avoid ambiguity.

Line 235: I do not believe the authors present robust evidence to support the claim that there is antigenic divergence between these 2 OROV strains. Data are from n=3 animals.

We thank the reviewer for this comment and agree that the original wording overstated the strength of the evidence. In the revised manuscript, we have removed claims of antigenic divergence between BeAn19991 and OROV 240023. We now describe the data more conservatively as differences in neutralizing antibody responses and cross-neutralization profiles, without inferring antigenic divergence.

Line 246-249: as noted elsewhere in this review, I do not agree with these statements.
We thank the reviewer for this feedback. We agree that our original wording may have overstated the strength of our conclusions. To address this, we have revised the text to use more cautious language, framing our observations as suggestive rather than definitive and highlighting the need for additional mechanistic work.

Figure 5 would benefit from a survival curve. Additionally, what was the nAb titer in the animals at the timepoint in which they were challenged?
We thank the reviewer for this suggestion. We respectfully note that a survival curve would be of limited additional value for this experiment, as the data are derived from a defined time-course study in which animals were euthanized at predetermined timepoints for tissue collection rather than followed longitudinally to survival endpoints. Mortality was therefore not an experimental outcome variable but occurred incidentally within the context of scheduled necropsies. For this reason, survival was described qualitatively in the text rather than presented as a Kaplan-Meier analysis. Regarding neutralizing antibody titers at the time of challenge, we agree that this information would be informative. However, sera were not collected immediately prior to challenge in this experiment, precluding direct measurement of neutralizing antibody titers at that timepoint.

Line 281: the statement that the capacity for OROV for maternal-to-fetal transmission has been demonstrated needs a citation. It was this reviewer's understanding that data were limited, with reports of suspected Oropouche-associated vertical transmission, miscarriage, and congenital abnormalities.
We thank the reviewer for this comment. We have added appropriate references to support this statement in the revised manuscript.

Line 293-294: This could be tested experimentally but without competition data there is not robust evidence to support this statement.
We thank the reviewer for this point. We agree that robust evidence for competitive advantage in natural transmission cycles would require direct competition assays, which were not performed in this study. To avoid overstating our findings, we have removed this sentence from the text.

Line 307: neutralization data do not support the statement that there are antigenically distinct strains.
We thank the reviewer for this comment. In the revised manuscript, we have removed this statement and now describe the findings more conservatively as differences in neutralizing antibody responses and cross-neutralization profiles, without inferring antigenic distinction.

Line 426: MOI should have units associated with the value, e.g., PFU/cell or similar.
We thank the reviewer and have clarified in the Methods that MOI values are expressed as $TCID_{50}$ units per cell (rather than PFU/cell), based on titration of virus stocks by endpoint dilution.

Discussion: overall the discussion would benefit from a paragraph about related orthobunyaviruses and congenital infection.
We thank the reviewer for this suggestion. As requested, we have expanded the Discussion to incorporate comparisons with related orthobunyaviruses (e.g., Akabane virus, Schmallenberg

virus, Shuni virus), highlighting their known congenital and CNS outcomes. This provides a stronger contextual framework for interpreting OROV pathogenesis in our model. (Revised text: lines 1118-1129).

REVIEWERS' COMMENTS

Reviewer #1 (Remarks to the Author):

The manuscript has been revised in response to most of the points raised and has improved substantially.

However, several issues still need to be addressed:

- To better assess how suitable the mouse model is for studying the pathogenesis of this virus, the authors should discuss available data from related viruses, such as AKAV or SBV, for which both mouse and target-host data exist. This would help readers to better judge the value and limitations of mouse models and their transferability to the natural host species.

We thank the reviewer for this suggestion. We have added text to the Discussion placing our murine model in the context of experimental systems used for related orthobunyaviruses, including Akabane and Schmallenberg viruses. These comparisons highlight that while mouse models provide important mechanistic insight, they often do not fully recapitulate disease observed in natural ruminant hosts, underscoring both the utility and limitations of murine systems for studying orthobunyavirus pathogenesis.

- Comparison with prior experiments, particularly OROV studies in mouse models (including the IFNAR-/- model), should be provided. This point is still insufficiently addressed.

We thank the reviewer for this suggestion. We have revised the Discussion to explicitly place our findings in the context of prior OROV mouse studies, including IFNAR^{-}/$^{-}$ models.

- Although additional sequencing data have now been included, whole-genome sequences e.g. from the fetal tissues are still missing. Providing these data remains recommended, as they could offer important insight into the role of fetal infection.

We thank the reviewer for this suggestion. We agree that whole-genome sequencing of viruses from fetal tissues could provide valuable insight into potential adaptation during vertical transmission. However, this analysis was not feasible within the current study due to the limited viral material recovered from fetal tissues and the short infection window, which would likely preclude robust detection of within-host variation. Since the current study is focused on establishing and characterizing a model of OROV vertical transmission, we will consider this an important direction for future investigation.

- The role of innate immunity is demonstrated in the mouse model. It should therefore be clearly stated that these findings cannot automatically be extrapolated to human infections (see lines 392-400).

We thank the reviewer for this comment. We have revised the Discussion to more clearly distinguish the role of innate immune responses from broader limitations of the murine model.

- Lines 747-749: a supporting reference is missing for the statements.

We thank the reviewer for pointing this out. We have now included references supporting our statements.

- An additional ethics-related clarification is needed. In line 547, it is stated that all animals "succumbed to disease." Please clarify that humane endpoints were applied and that animals were euthanized once these endpoints were reached.

We thank the reviewer for this important clarification. We have revised the manuscript to specify that animals were monitored closely and euthanized upon reaching predefined humane endpoints. This has been included in the ethics section of our methods.

Reviewer #2 (Remarks to the Author):

The revised manuscript has adequately addressed the concerns raised, including adding additional data as needed, tempering conclusions that were overstated, clarifying aspects of experiments that were originally unclear, and added appropriate controls and statistical analyses.

The results are of interest to the field, and the results and conclusions are now supported by the data.

We thank the reviewer for their careful evaluation of our revised manuscript and for their positive feedback. We are pleased that the additional data, clarifications, and revisions have addressed the concerns raised and that the results and conclusions are now considered well supported.

Reviewer #3 (Remarks to the Author):

The authors addressed most of my previous concerns. However, a few still remain:

Line 72-73: some references should be included here to support these statements.

We thank the reviewer for pointing this out. We have now included references supporting our statements.

Line 88: the references cited here do not directly address changes in Culicoides range, etc.

We thank the reviewer for this comment. We have revised the text to more accurately reflect the scope of the cited literature regarding environmental and climate-related influences on vector ecology.

Line 90: "appears to be evolving" is contradictory to the findings presented here. The authors could say something like the clinical profile associated with Oropouche virus infection has expanded with the recent outbreaks to include pregnancy complications, neurological manifestations, etc. However, there are many reasons why this might be occurring, only one of which is that the virus has evolved new properties, so I would be careful how you frame this.

We thank the reviewer for this helpful comment. We have now revised the text as suggested.

Line 139: I defer to the editor here but I would remove "novel" from the title and the text. There is nothing inherently novel with the mouse model since it is C57BL/6. The novelty stems from

assessing the pathogenic potential of OROV in the setting of pregnancy not necessarily in the model used.

The word “novel” has now been removed from the title and the text.

Line 306-309: can you include the differences between virus strains in a table in the supplementary files?

We thank the reviewer for this suggestion. The sequence differences between the virus strains used in this study have been previously reported and are available in the original publications and associated GenBank entries. To avoid redundancy, we have not included such a table in our manuscript.

Line 93: the statement about IFN-I as a key barrier is not accurate. Since you mated WT sires with IFNAR1-/- dams the placentas and fetuses will have one intact Ifnar1 allele and thus maintain IFN signalling. It might not be as robust as with 2 WT animals mated but more experiments would be required to tease this apart.

We thank the reviewer for this important point. We agree that in the IFNAR$^{-/-}$ × WT cross, fetal and placental tissues retain one functional Ifnar1 allele and therefore maintain partial type I interferon responsiveness. Our intention was not to imply that type I interferon signaling is completely absent at the maternal-fetal interface. We have revised the text to clarify that the primary effect of IFNAR deficiency in this model is enhanced systemic viral replication in the dam, which in turn increases the likelihood of placental infection and vertical transmission.

Line 436: replace “live viruses” with something like “these virus isolates were not available from public repositories” or something similar.

We thank the reviewer for this suggestion and have revised the text accordingly.

Fig 6. Can the authors please clarify? Neutralization was assessed using immune sera collected at 5dpi (pregnant) and 14dpi (nonpregnant) and conclude that there is poor seroconversion against the homologous virus. However, these timepoints are rather early (particularly the 5dpi) to see evidence of robust seroconversion. Typically, one would need to wait 21-28 days.

We thank the reviewer for this important point. We agree that the timepoints examined (5 dpi and 14 dpi) represent early stages of the humoral response and are not optimal for assessing fully developed seroconversion. Our intention was to compare relative neutralizing activity at these defined timepoints rather than to assess mature antibody responses. We have therefore revised the text to avoid the term “seroconversion” and instead describe these data as early neutralizing antibody responses.

Line 701: is this timeframe normal for mice for maternal transfer of Ab? Can you include a citation?. In humans and monkeys you have a window of about 3-6 months.

We thank the reviewer for pointing this out. We have now included references to support this timeframe.

Response to Reviewer comment about RT-qPCR limit of detection: The authors responded with the following: “The apparent discrepancy arises because points plotted below the indicated LoD represent samples with detectable signal that fell below the quantitative range of our standard

curve; these are shown for transparency but should be interpreted as below the quantitative threshold." Based on this explanation is this not the "limit of quantification" which is different from the LoD. If you look at the MIQE guidelines (PMID: 19246619), the limit of detection is defined "as the lowest concentration at which 95% of the positive samples are detected. In other words, within a group of replicates containing the target at concentrations at the LoD, no more than 5% failed reactions should occur." The lower limit of quantification is harder to define. As a heuristic I think it is okay to say that LLoQ is the same as the LoD, but I'm not sure it is "transparent" to show these because they should not be considered positive.

We thank the reviewer for this thoughtful comment and agree that the distinction between limit of detection (LoD) and lower limit of quantification (LLoQ) is important. In our analysis, values plotted below the indicated threshold represent samples with a detectable signal that falls below the quantitative range of the assay. These values were included to reflect the full dataset and to distinguish between a complete absence of signal and low-level detection. We do not interpret these values as robust positive or quantitative measurements, but rather as indicative of a signal at or near the detection threshold. We believe that the inclusion of these data provides additional context and transparency without affecting the overall conclusions of the study.
